# The Effect of Branching on the One‐ and Two‐Photon Absorption, Cell Viability, and Localization of Cationic Triarylborane Chromophores with Dipolar versus Octupolar Charge Distributions for Cellular Imaging†

**DOI:** 10.1002/chem.201902461

**Published:** 2019-09-17

**Authors:** Stefanie Griesbeck, Evripidis Michail, Florian Rauch, Hiroaki Ogasawara, Chenguang Wang, Yoshikatsu Sato, Robert M. Edkins, Zuolun Zhang, Masayasu Taki, Christoph Lambert, Shigehiro Yamaguchi, Todd B. Marder

**Affiliations:** ^1^ Institut für Anorganische Chemie, and Institute for Sustainable Chemistry, & Catalysis with Boron Julius-Maximilians-Universität Würzburg 97074 Würzburg Germany; ^2^ Institut für Organische Chemie Julius-Maximilians-Universität Würzburg 97074 Würzburg Germany; ^3^ Institute of Transformative Bio-Molecules Nagoya University Nagoya Japan; ^4^ Department of Pure & Applied Chemistry University of Strathclyde Glasgow UK; ^5^ State Key Laboratory of Supramolecular Structure and Materials College of Chemistry Jilin University Qianjin Street Changchun P. R. China

**Keywords:** boranes, cell imaging, fluorescence, lysosome, two-photon excited fluorescence

## Abstract

Two different chromophores, namely a dipolar and an octupolar system, were prepared and their linear and nonlinear optical properties as well as their bioimaging capabilities were compared. Both contain triphenylamine as the donor and a triarylborane as the acceptor, the latter modified with cationic trimethylammonio groups to provide solubility in aqueous media. The octupolar system exhibits a much higher two‐photon brightness, and also better cell viability and enhanced selectivity for lysosomes compared with the dipolar chromophore. Furthermore, both dyes were applied in two‐photon excited fluorescence (TPEF) live‐cell imaging.

## Graphical abstract

**Dipole versus octupole**: Branching of a cationic triarylborane chromophore leads to a cooperative increase in the two‐photon absorption cross‐section and brightness, as well as higher selectivity towards lysosomes and better cell viability. The octupolar dye was demonstrated to be an ideal candidate for two‐photon excited fluorescence imaging of lysosomes in live cells.

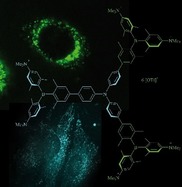

## Introduction

Triarylboranes have aroused much interest in materials applications in the last few decades.^1^ Due to the empty p_*z*_‐orbital of the three‐coordinate boron atom, they are used as strong π‐acceptors (A), when conjugated to a π‐donor (D). In 1972, Williams and co‐workers at Kodak reported the photophysical properties of several *para*‐substituted aryldimesitylboranes.^2^ Although the absorption maxima were only slightly affected by solvent polarity, the fluorescence maxima showed a large bathochromic shift with increasing solvent polarity. This suggests a small dipole moment in the ground state and a large increase in the dipole moment in the first excited singlet state, which can be better stabilized in polar solvents. Thus, triarylboranes are excellent π‐acceptors in intramolecular charge‐transfer compounds, for example, in dipolar chromophores, because they show highly solvatochromic emission.^3^ Furthermore, excitation‐induced charge‐transfer properties increase the two‐photon absorption (TPA) probability.^4^ Therefore, three‐coordinate boron compounds have great potential for TPA^5^ and other nonlinear optical (NLO) applications.^6^

**Scheme 1:**
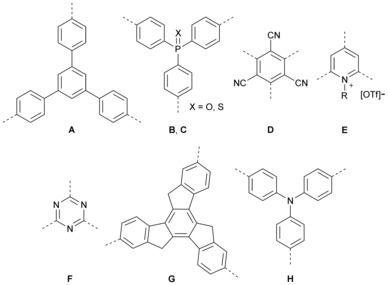
Schematic drawing of the different cores **A**–**H**.

Degenerate two‐photon absorption is a third‐order nonlinear optical process, which involves the simultaneous absorption of two photons.^7^ Given that the final state is reached by two‐photon absorption via a virtual state, the energy of the photons is half of the actual energy gap between the ground and excited states. For typical chromophores, this means near‐infrared light is required, which is highly desirable for fluorescence microscopy of live cells and tissues, because of the deeper tissue penetration of these longer wavelength photons. There are three characteristic structural motifs known for efficient organic TPA dyes, namely dipole (D–A), quadrupole (d–π–D, A–π–A) or octupole (D–A_3_, A–D_3_). Attention has progressively moved from well‐known push‐pull systems to quadrupoles and octupoles, because they exhibit larger TPA cross‐sections (*σ*_2_). Quadrupolar dyes are the most studied for two‐photon excited fluorescence, and we have also studied them for live‐cell imaging.^8^ In this paper, we concentrate on the differences between dipolar and octupolar triarylborane dyes.5f Properly speaking, the latter are three dipoles connected by a trigonal core which can display cooperative (>3×*σ*_2_(dipole)), additive (3×*σ*_2_(dipole)) or suppressive (<3×*σ*_2_(dipole)) effects of the branching.7a Prasad first demonstrated the cooperative effect with a triphenylamine donor core, branched with three 2‐phenyl‐5‐(4‐*tert*‐butylphenyl)‐1,3,4‐oxadiazole acceptors.^9^ Further studies of octupolar systems showed that the effect of branching depends on the nature and strength of the coupling between the three arms and the nature of the core.^10^ Although triphenylbenzene **A**,^11^ triphenylphosphine oxide **B**, and triphenylphosphine sulfide **C** 12 as a core exhibit only an additive effect, tricyanobenzene **D**,^13^ pyridinium **E**,^14^ *s*‐triazine **F**,^15^ truxene **G**,^16^ and triphenylamine **H** 5g, 9, 17 showed highly cooperative behavior (Scheme 1). To understand the influence of the coupling, several models were investigated.17e, 18 The Frenkel exciton model, in which only electrostatic interactions of the dipole units are considered, led to qualitatively good results and a correct order of the excited states for octupolar compounds but, given that the donor or acceptor in the core is shared by the three arms, this model does not provide quantitative estimations of nonlinear properties. As soon as the coupling becomes stronger, and the charge is more delocalized over the three branches, electron‐vibration interactions and/or solvent effects must be taken into account. Therefore, essential‐state models or correlated quantum‐chemical approaches are more accurate. Fang and co‐workers compared octupolar dyes with a triphenylamine core, a conjugated central moiety, and a triethanolamine core, which is nonconjugated.^19^ Given that the nonconjugated moiety is not able to couple electronically, and no cooperative enhancement was observed, it was demonstrated that electronic coupling still plays the major role, whereas the vibronic coupling is often overrated. Therefore, the Frenkel exciton model gives a qualitatively good approximation of the two‐photon absorption enhancement. Müllen classified the electronic coupling constant *V* as being “small” (≤0.05 eV), “increased” (0.05 eV≤*V*≤0.15 eV), or “strong” (0.15 eV≤*V*≤0.25 eV).18b Even though the weak coupling only leads to additive enhancement and no interaction in the excited states, increased coupling yields cooperative enhancement. However, the interaction between the branches in the excited state is not dominant, because the excitation localizes on a dipolar chromophore branch prior to emission.^20^ The strong coupling case is more complex, and leads to strong enhancement, as the exciton is completely delocalized and emission occurs from the entire system.

Only a few octupolar dyes have been explored for two‐photon excited fluorescence imaging (TPEF) thus far. In 2007, the first octupolar dye, with a triphenylamine core branched to three pyridinium acceptor moieties for solubility and electron‐withdrawing strength, was reported to have a two‐photon absorption cross‐section of 700 GM in glycerol.^21^ Unfortunately, the two‐photon absorption cross‐section was not measured in buffer because the dye is almost non‐emissive (*Φ*_f_=0.02, *Φ*_f_: fluorescence quantum yield), but it acts as a turn‐on sensor when binding to DNA occurs. In fixed CHO‐K1 cells, the dye was found to localize in the nucleus and bind selectively to DNA. This dye was further improved by the same group by using *N*‐methyl benzimidazolium moieties as the acceptor units, leading to a higher fluorescence quantum yield and DNA affinity.^22^ Blanchard‐Desce and co‐workers also examined octupolar systems for TPEF. In 2011, their first report involved the preparation of nanoparticles with molecules containing a triphenylamine core and 2‐formylthiophene as the acceptor unit, but these were found to aggregate very rapidly and deposit in small blood vessels,^23^ thereby hindering blood flow, leading to the death of the tadpole they were studying. Two years later, they reported two symmetric octupolar dyes for cell imaging,^10^ both of which have a triphenylamine core and SO_2_CH_2_CH_2_OH as the peripheral acceptor for improved solubility. The donor and acceptor groups were connected by phenyl–ethynyl and phenyl–vinyl bridges. The TPA brightness (*σ*_2_ *Φ*_f_) of the two compounds in ethanol solution were found to be 250 and 268 GM, respectively, and TPEF images showed the localization of the dyes in the cytoplasm of HEK 293 cells. Another octupolar dye that selectively stains the cytoplasm was reported by Tian and co‐workers.^24^ This dye bears a triphenylamine core and bis‐cyano‐substituted isophorones as acceptors. The Yang group sensed H_2_S with a Cu^II^‐cyclen‐substituted triarylborane.^25^ They reported cell‐membrane permeability and a preferential distribution at mitochondria,^25^ whereas the same compound, without Cu^II^ binding, was used one year later to stain nucleoli and cytoplasm.^26^ However, a two‐photon brightness of only 30 GM in DMSO was measured for this compound. Very recently, an octupolar dye was reported which stains nucleoli as well as the nuclear membrane, nuclear matrix, nuclear pore and the cytoplasm, while binding to RNA.^27^ This dye comprises a triarylborane acceptor core branched by three piperazine donors, and has a two‐photon brightness of 90 GM. Attaching multiple cyclic arginine–glycine–aspartic acids to this compound leads to accumulation at integrin α_v_β_3_, which is overexpressed in cancer cells.^28^

Thus far, there have been no studies on the difference between dipolar and octupolar systems in cell imaging, comparing their selectivity and toxicity. Therefore, we synthesized a dipolar dye with a triphenylamine donor and a triarylborane acceptor. Given that triphenylamine is an efficient core for cooperative TPA enhancement (see above), we used this core for our octupolar system and connected it to three triarylborane acceptors. Our triarylborane acceptors are substituted with trimethylammonio groups to achieve good water solubility.^29^ We report herein a comparison of the linear and nonlinear optical properties as well as the differences between the two dyes when used for live‐cell fluorescence imaging.

## Results and Discussion

### Synthesis

**Scheme 2:**
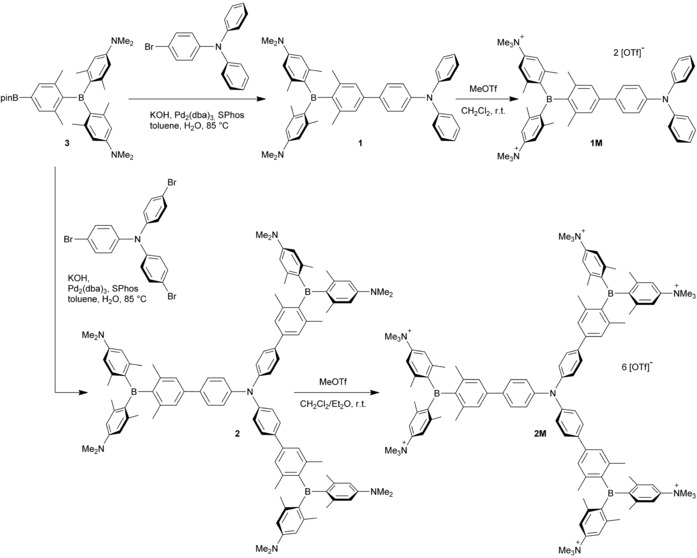
Synthesis of the target molecules **1M** and **2M**.

The neutral dyes **1** and **2** were prepared via Suzuki–Miyaura cross‐coupling reactions. For details of the synthesis and characterization of all compounds see the Supporting Information. Thus, the borylated triarylborane **3**, which was previously reported by our group,8a and 4‐bromo‐*N*,*N*‐diphenylaniline or tris(4‐bromophenyl)amine were coupled using Pd_2_(dba)_3_ as the catalyst, SPhos (2‐dicyclohexylphosphino‐2′,6′‐dimethoxybiphenyl) as the ligand, and potassium hydroxide as the base. To strengthen the acceptor ability of the boron center, and to enhance water solubility, the neutral dyes were methylated with methyl triflate to yield the cationic dyes **1M** and **2M** in almost quantitative yields (Scheme 2). Unfortunately, neither dye was soluble in pure water, but they could be dissolved upon addition of 0.5 % DMSO, with no nanoparticles observable by dynamic light scattering (DLS) measurements.

### Linear optical properties of and TD‐DFT calculations on neutral chromophores 1 and 2

**Figure 1:**
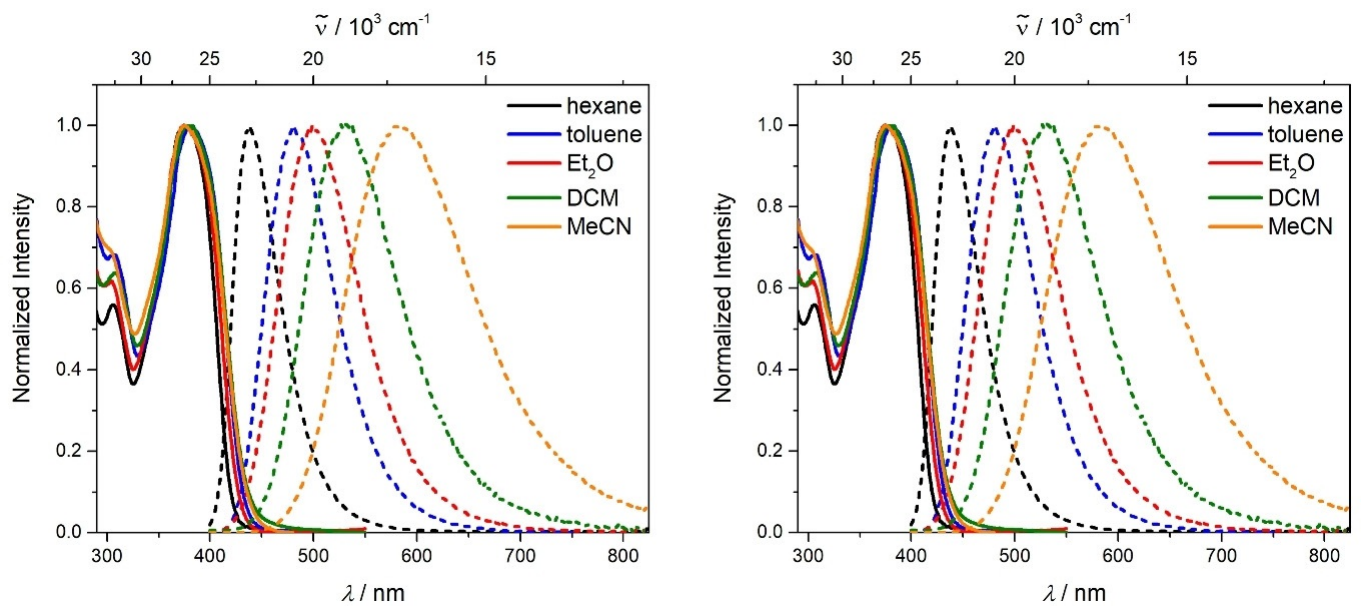
Absorption (solid lines) and emission spectra (dashed lines) of **1** (left) and **2** (right) in various solvents (hexane: black, toluene: blue, diethylether: red, DCM: green, MeCN: orange) at room temperature (*λ*_ex_=λmaxabs ).

The absorption spectra of **1** and **2** display one broad absorption band at wavelengths greater than 300 nm, which shows almost no solvatochromism (Figure 1 and Table 1). In compound **1** the absorption maximum occurs at about 380 nm (S_2_←S_0_ transition) with a shoulder around 392 nm (S_1_←S_0_ transition), which is exactly the absorption maximum of **2** (See Figure S1 in the Supporting Information for an enlarged display of the absorption band). We performed DFT (B3LYP/6‐31G(d) level of theory) and TD‐DFT (CAM‐B3LYP/6‐31G(d)) calculations in the gas phase for both compounds **1** and **2** to obtain a better understanding of the absorption spectra. The HOMO is localized on the triphenylamine, and HOMO−1 (and HOMO−2, HOMO−3 for **2**) are localized on the dimethylamine (Figure S2). The HOMO and HOMO−1 are isoenergetic for compound **1**, whereas in compound **2** the HOMO is slightly higher in energy than the isoenergetic HOMO−1, HOMO−2, and HOMO−3. The TD‐DFT calculations of the S_1_←S_0_ transition in the neutral compounds **1** and **2** show that the short‐range charge transfer (CT) from the dimethylamino groups to the boron atom predominates over the long‐range CT from the triphenylamine to the boron center, even though the HOMO is localized on the triphenylamine. In the geometry optimized structures, the phenyl group(s) of the triphenylamine involved in the link(s) between N and B and the xylyl group(s) of the boron moiety have a torsion angle of 35° in both molecules (**1** and **2**), which hinders efficient long‐range charge transfer. However, the higher energy transitions, S_2_←S_0_ and S_3_←S_0_, of **1** have increasing contributions from the long‐range CT because they have greater HOMO contributions (Table 2).

**Table 1:** Photophysical data for the compounds **1** and **2** in various solvents.

	Solvent	*λ*_abs_ [nm]	*ϵ* [m ^−1^ cm^−1^]	*λ*_em_ [nm]	Stokes shift [cm^−1^]	*Φ*_f_	*τ* [ns]	*τ*_0_ [ns]	*k*_r_ [10^8^ s^−1^]	*k*_nr_ [10^8^ s^−1^]
**1**	hexane	375	58000	438	3800	0.13	1.6	12.3	0.8	5.5
	toluene	383		481	5300	0.21	3.1	14.8	0.7	2.5
	Et_2_O	375		499	6600	0.22	4.7	21.4	0.5	1.6
	DCM	383		527	7100	0.31	8.5	27.4	0.4	0.8
	MeCN	376		583	9400	0.12	5.0	41.7	0.2	1.8
**2**	hexane	391	186000	439	2800	0.17	1.4	8.2	1.2	5.9
	toluene	396		481	4700	0.23	3.2	13.9	0.7	2.4
	Et_2_O	390		503	5800	0.23	4.9	21.3	0.5	1.5
	DCM	394		536	6700	0.33	8.5	25.8	0.4	0.8
	MeCN	391		588	8600	0.13	4.8	36.9	0.3	1.8

**Table 2:** TD‐DFT calculations on **1** and **2** in the gas phase.

	State	Symmetry	*E* [eV]	*λ* [nm]	*f*	Major (>10 %) contributions^[a]^
**1**	S_1_	A	3.56	349	0.327	H−1→L (86 %)
	S_2_	A	3.75	331	0.708	H−3→L (13 %), H−2→L (41 %), H→L (32 %)
	S_3_	A	4.15	299	0.320	H−2→L (42 %), H→L (23 %), H→L+1 (24 %)
**2** *C*_3_	S_1_	E	3.56	348	0.161	H−3→L (27 %), H−2→L+1 (13 %), H−2→L+2 (25 %), H−1→L (13 %)
	S_2_	E	3.56	348	0.161	H−3→L+1 (27 %), H−2→L (13 %), H−1→L+1 (13 %), H−1→L+2 (25 %)
	S_3_	A	3.56	348	0.611	H−3→L+2 (29 %), H−2→L (28 %), H−1→L+1 (28 %)
**2** *C*_1_	S_1_	A	3.56	348	0.161	H−3→L (12 %), H−2→L (11 %), H−1→L+1 (19 %), H−1→L+2 (26 %)
	S_2_	A	3.56	348	0.161	H−3→L (21 %), H−3→L+1 (15 %), H−2→L+1 (14 %), H−2→L+2 (28 %)
	S_3_	A	3.56	348	0.611	H−3→L+2 (27 %), H−2→L (20 %), H−1→L+1 (19 %)

[a] H: HOMO, L: LUMO.

**Figure 2:**
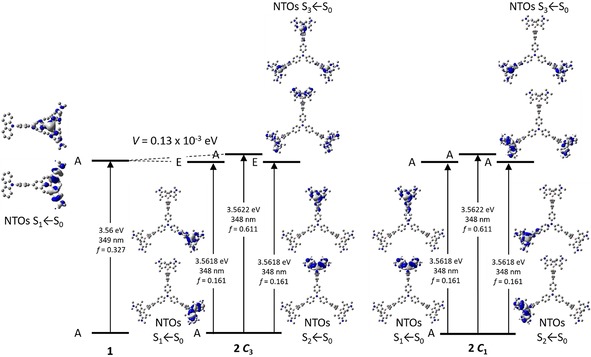
Excited‐state splitting of **2** in *C*_3_ and *C*_1_ symmetry with respect to **1**. The natural‐transition orbitals (NTOs) of S_1_←S_0_, S_2_←S_0_ and S_3_←S_0_ are depicted from TD‐DFT calculations in the gas phase.

To investigate the coupling between the three branches of **2**, we use the exciton‐coupling model. Coupling of the three excited states in *C*_3_ symmetry leads to two degenerate excited states (S_1_ and S_2_), which are stabilized by the coupling constant *V* and have E symmetry, and one excited state (S_3_), which is destabilized by 2 *V* and has A symmetry (Figure 2). Given that excitation from S_0_ (A symmetry) is only allowed to S_1_ and S_2_ (E symmetry), one might observe the coupling constant *V* from the shift of the S_1_←S_0_ absorption bands. Comparing the calculated S_1_←S_0_ absorption band of **1** to that of **2** (optimized gas‐phase geometry for **1** and **2** and also *C*_3_‐symmetrized geometry for **2**) gives a negligible difference; therefore, coupling between the three arms is very small or non‐existent and the coupling constant *V* is ≈0.00 eV.18b The experimentally determined absorption bands at 392 (the shoulder in the absorption spectrum of **1**) and 391 nm (the absorption maximum of **2**) confirm this. Furthermore, the extinction coefficient *ϵ*=186000 m ^−1^ cm^−1^ measured for **2** being approximately 3 times that of **1** (*ϵ*=58000 m ^−1^ cm^−1^) shows additive behavior, because the three individual branches in **2** can be excited, but the emission occurs from a localized single branch. That is why the emission spectra as well as the fluorescence quantum yields and lifetimes of **1** and **2** are similar (Table 1). The emission maximum redshifts with increasing solvent polarity, because the CT excited state becomes more stabilized, which is well known for D–A compounds. However, fluorescence quantum yields and lifetimes do not follow the expected dependence on solvent polarity. The quantum yields increase from nonpolar to polar solvents, whereas the nonradiative decay rates *k*_nr_ decrease. This is exactly the opposite of what would be expected from the energy‐gap law.^30^ Usually, the nonradiative decay rate *k*_nr_ increases and, therefore, the quantum yield decreases. The fluorescence lifetimes become longer with increasing solvent polarity, whereas the radiative decay rates *k*_r_ are in qualitative accordance with the Strickler–Berg equation,^31^ decreasing with decreasing emission energy. Furthermore, in acetonitrile (MeCN), both compounds do not follow the aforementioned trend, because the quantum yields are decreased and fluorescence lifetimes are shorter compared with dichloromethane (DCM) solutions. This behavior was observed previously for nitrogen‐donor–boron‐acceptor compounds3c, 32 and has its origin in symmetry breaking in the excited state. The symmetry breaking is more enhanced in polar solvents than in nonpolar solvents, leading to the unusual solvent behavior seen above.^33^ As the two short‐range CTs in compound **1** are arranged in *C*_2_ symmetry, the symmetry can break in the excited state, resulting in the observed unusual behavior of the fluorescence quantum yields and lifetimes in polar solvents. The long‐range CT is parallel to the *C*_2_ axis and, therefore, would not show symmetry breaking, and hence, no solvatochromism. In the branched compound **2**, the short‐range CT is the most dominant. In *C*_3_ symmetry, as well as in *C*_1_ symmetry, we do not observe coupling between the three subchromophore branches as the exciton coupling constant *V* between the three arms is negligibly small, being 0.13×10^−3^ eV (Figure 2). This is not astonishing because the triphenylamine core does not take part in the transitions. Therefore, chromophore **2** can be considered to be comprised of three independent subchromophores, each directly analogous to **1**. Thus, **2** exhibits the same photophysical properties as **1**.

### Linear‐optical properties of and TD‐DFT‐calculations on cationic chromophores 1M and 2M

**Figure 3:**
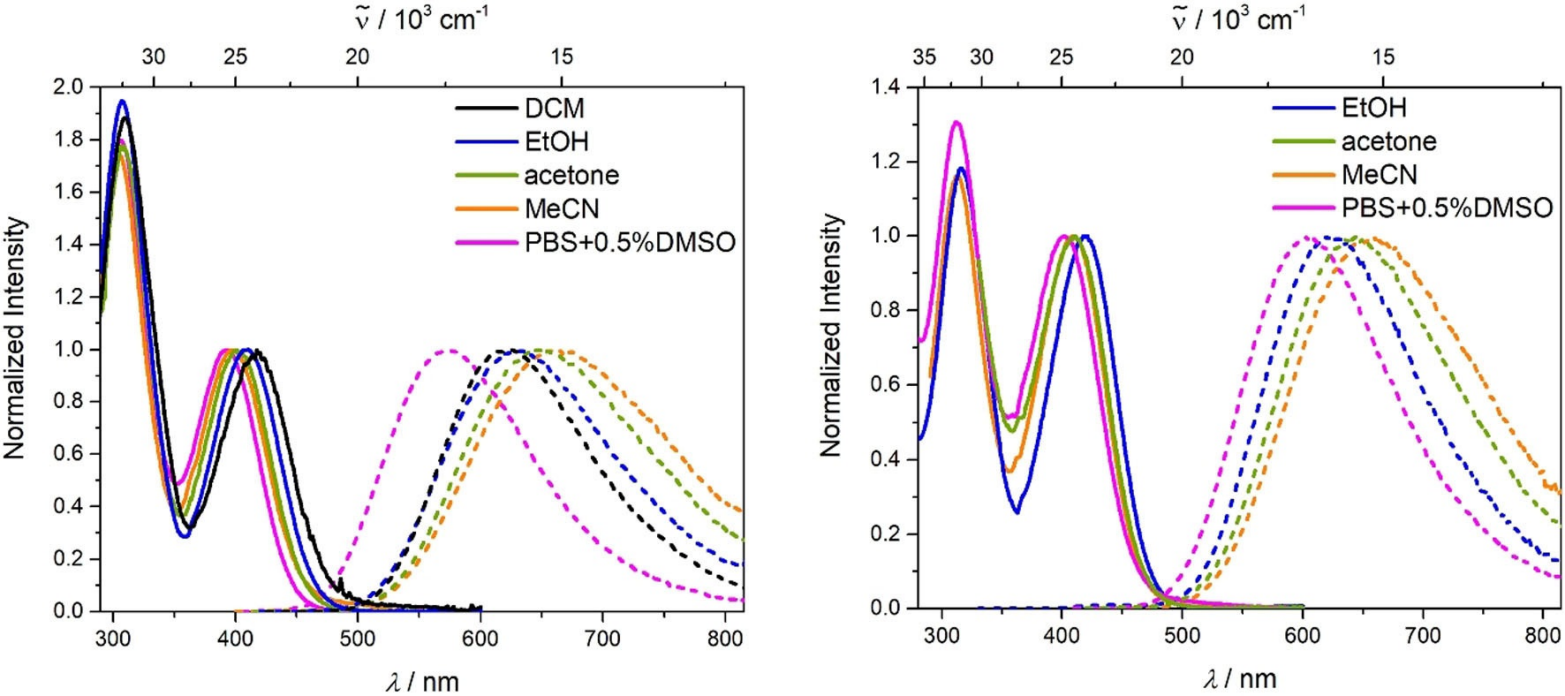
Absorption (solid lines) and emission spectra (dashed lines) of **1M** (left) and **2M** (right) in various solvents (DCM: black, ethanol: blue, acetone: green, MeCN: orange, PBS+0.5 % DMSO: pink) at room temperature (*λ*_ex_=λmaxabs >350 nm).

**Figure 4:**
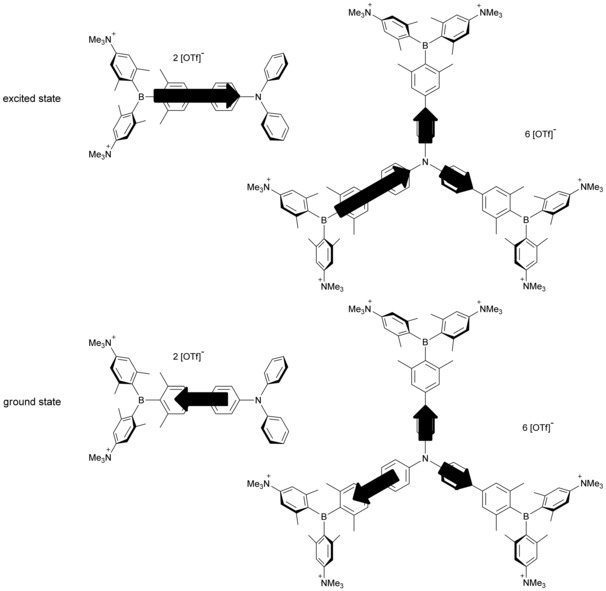
Schematic representation of the ground‐ and excited‐state dipole moments of **1M** and **2M**. The length of the arrows is not to scale with the absolute values of the dipole moments.

Upon methylation of all dimethylamino groups in the neutral dyes **1** and **2**, the photophysical properties of the chromophores change completely. The absorption spectra of **1M** and **2M** exhibit two bands (Figure 3). Although the higher energy absorption bands at approximately 305 and 314 nm, respectively, are almost solvent independent, the low‐energy absorption band is significantly hypsochromically shifted with increasing solvent polarity. Within the limited range of solvents in which the compounds are readily soluble, the absorption maxima increase in energy with increasing solvent dipole moment, rather than increasing solvent orientation polarizability ((*ϵ*−1/2 *ϵ*+1)−(*n* ^2^−1/2 *n* ^2^+1), where *ϵ* is the dielectric constant and *n* is the refractive index of the solvent), as used for Lippert–Mataga plots. Strong deviations in the solvatochromic behavior might occur due to differences in ion pairing between di‐cation **1M**, hexa‐cation **2M**, and their counterions in the various solvents. Given that compound **2M** shows solvatochromism in its absorption, and thus possesses a non‐zero dipole moment, it must undergo symmetry breaking in the ground state to a symmetry lower than *C*_3_. Comparing the absorption spectra of **1M** and **2M** in EtOH shows a bathochromic shift of 704 cm^−1^_._ Using the exciton‐coupling model (see above) a coupling constant of 0.09 eV was calculated. The branching leads to a delocalization and therefore a redshifted absorption.5g, 17c Given that this is “increased” but not “strong” coupling, as classified by Müllen (see above), the extinction coefficient shows approximately additive behavior (Table 3). Furthermore, the excitation localizes on a dipolar chromophore branch prior to emission. Therefore, the compound also has an excited‐state dipole moment. Thus, the emission maxima are bathochromically shifted, except for the PBS (phosphate‐buffered saline) +0.5 % DMSO solution in which special ion–ion interactions might occur. Given that the absorption is hypsochromically shifted, and the emission is bathochromically shifted in more polar solvents, an inversion of the dipole moment upon excitation occurs. This contrasts with the short‐range CT transition of compounds **1** and **2**, in which the absorption is not solvatochromic and the emission shows positive solvatochromism, which implies that the dipole moment retains its original direction. The charge‐transfer behavior in the ground and excited states of dipolar and trigonal boron chromophores similar to the neutral dyes **1** and **2** and the cationic dyes **1M** and **2M** was reported by Lambert and co‐workers in 2006.32a In the less‐hindered neutral chromophores **1** and **2** (one xylyl group between the nitrogen and the boron atoms), the ground‐state polarization is dominated by mesomeric effects, leading to a charge‐separated quinoidal contribution to the structure with a partial negative charge on the boron atom and a partial positive charge on the nitrogen atom, which increases after charge‐transfer upon excitation. In contrast, chromophores **1M** and **2M** have less effective π‐conjugation between the two boron atoms as the xylyl and the phenyl group are twisted. Therefore, the ground‐state polarization is mainly influenced by inductive effects, that is, boron as a σ‐donor and nitrogen as a σ‐acceptor. This leads to an inversion of the direction of the ground‐ versus excited‐state dipole moments. Given that the solvatochromism is more pronounced in the emission than the excitation, $\vec{\mu }$_*e*_ must be larger than $\vec{\mu }$_g_ for both cationic compounds **1M** and **2M**. The value of $\vec{\mu }$_g_ of **2M** must be smaller than $\vec{\mu }$_g_ of **1M**, because the two other branches also have a small contribution to the dipole moment, as illustrated in Figure 4. That this is the case is demonstrated by the smaller negative absorption solvatochromism observed for **2M** (shift of 524 cm^−1^ from EtOH to MeCN) than for the **1M** analogue (shift of 618 cm^−1^ from EtOH to MeCN). In comparison, the positive emission solvatochromism is more enhanced for **2M** than **1M**, resulting in a larger $\vec{\mu }$_e_ (−624 and −874 cm^−1^ from EtOH to MeCN, respectively).

**Table 3:** Photophysical data for the cationic compounds **1M** and **2M** in various solvents.

	Solvent	*λ*_abs_ [nm]	*ϵ* [m ^−1^ cm^−1^]	*λ*_em_ [nm]	Stokes shift [cm^−1^]	*Φ*_f_	*τ* [ns]	*τ*_0_ [ns]	*k*_r_ [10^8^ s^−1^]	*k*_nr_ [10^8^ s^−1^]
**1M**	DCM	417		622	7900	0.61	9.0	14.8	0.7	0.4
	EtOH	407		633	8800	0.31	4.9	15.8	0.6	1.4
	acetone	401		649	9500	0.23	4.7	20.4	0.5	1.6
	MeCN	397	19000	659	10000	0.19	3.4	17.9	0.6	2.3
	PBS+ 0.5 % DMSO	393		573	8000	0.09	10.6	117.8	0.08	0.9
**2M**	EtOH	419		624	7800	0.44	6.4	14.5	0.7	0.9
	acetone	411		643	8800	0.29	5.9	20.3	0.5	1.2
	MeCN	410	55 000	660	9200	0.26	5.0	19.2	0.5	1.5
	PBS+0.5 %DMSO	402		604	8300	0.15	7.4	49.3	0.2	1.1

However, the quantum yields are consistent with normal CT behavior for both compounds, following the energy‐gap law.^30^ They decrease for each compound with increasing solvent polarity, as the nonradiative decay rate *k*_nr_ increases, whereas the radiative decay rate *k*_r_ remains constant. In the PBS solution, the chromophores again behave differently; the fluorescence lifetimes increase, as the radiative decay rate *k*_r_ decreases dramatically, whereas the nonradiative decay rate *k*_nr_ is not as strongly affected. Branching leads to a slightly enhanced quantum yield as observed previously, due to the smaller nonradiative decay rates *k*_nr_.5g, 17c

**Figure 5:**
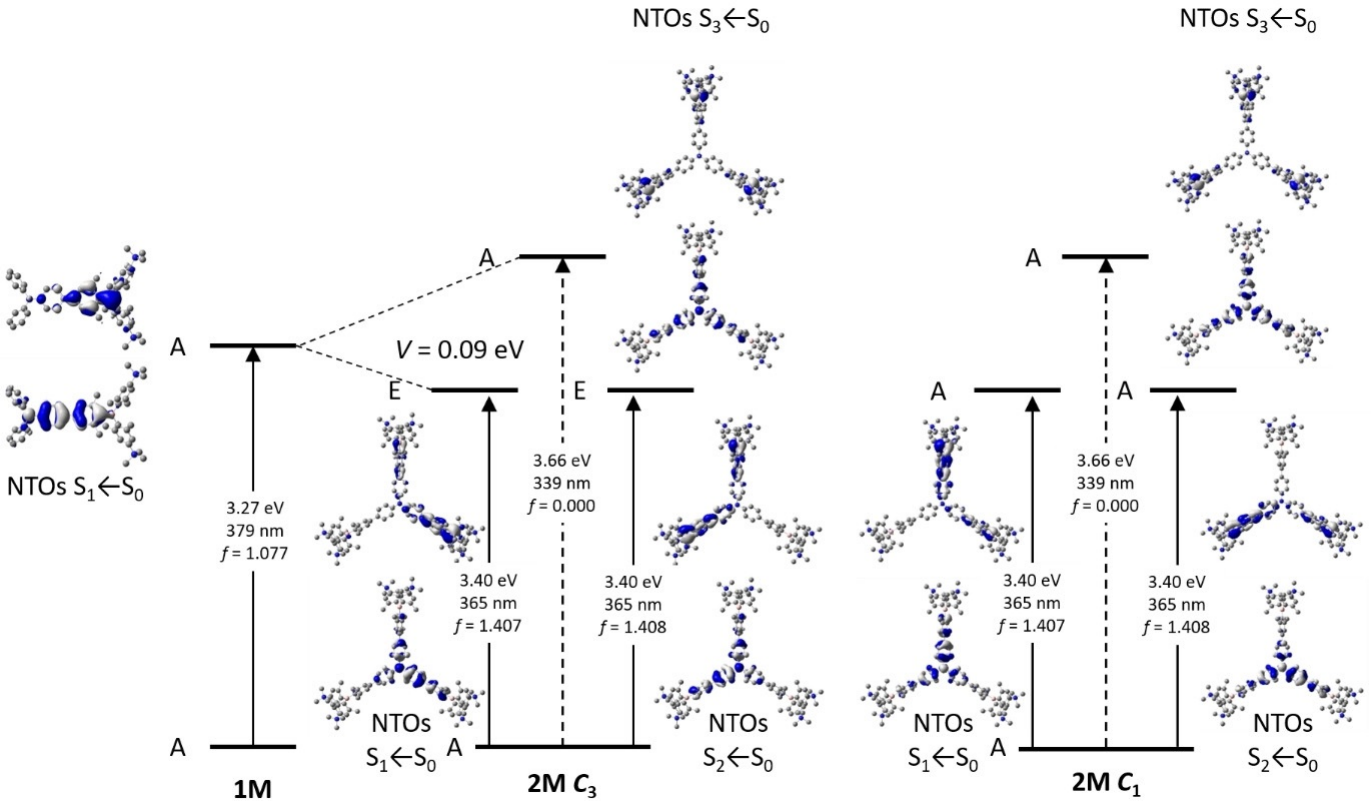
Excited‐state splitting of **2M** with respect to **1M**. The natural‐transition orbitals (NTOs) of S_1_←S_0_, S_2_←S_0_ and S_3_←S_0_ are depicted from TD‐DFT calculations in ethanol.

Methylation of compounds **1** and **2** destroys the short‐range CT between the lone pairs on the dimethylamino groups and the boron center and, concomitantly, the acceptor strength of the triarylborane units is enhanced by the inductive effect of the cationic trimethylammonio substituents. Therefore, in **1M** and **2M**, the transitions all involve CT from the triphenylamine to the boron atoms. DFT calculations on compounds **1M** and **2M** were carried out in the gas phase using the B3LYP functional in combination with the 6‐31G(d) basis set. The torsion angles between the phenyl groups of the triphenylamine and the xylyl groups of the boron moiety are reduced to 25° and 31° in **1M** and **2M**, respectively, compared with the neutral dyes **1** and **2**. Comparing the results from the TD‐DFT calculations (CAM‐B3LYP/6‐31G(d)) in the gas phase and EtOH show, especially for **1M**, a strong hypsochromic shift, because the CT is weaker in the polar solvents (Figure S4, Supporting Information). This can be seen in the natural transition orbitals (NTOs), which are more delocalized over the π‐system in EtOH than in the gas phase. In the octupolar compound **2M**, charge transfer from the central triphenylamine to one of the boron atoms of the three branches occurs. Given that the central triphenylamine contributes, the branches couple with each other. Because of the *C*_3_ symmetry, the S_1_ and S_2_ excited states are degenerate, stabilized relative to the S_1_ state of **1M** by the coupling constant *V*, and excitation from S_0_ is allowed (E symmetry, *f*=1.407), whereas S_3_ is destabilized by 2 *V* and S_3_←S_0_ is forbidden (A symmetry, *f*=0.000) (Table 4). From the exciton‐coupling model, the coupling constant *V* was calculated to be 0.09 eV, that is, one third of the energy difference between the TD‐DFT‐computed excited E and A symmetry states. This is exactly the same as the value obtained from the experimental shift between **1M** and **2M** in the UV/Vis absorption spectra. Both molecules show weak solvatochromism in their absorption spectra which indicates a small dipole moment in the ground state. However, given that the solvatochromism is quite pronounced in the fluorescence spectra, a moderate to large excited‐state dipole moment can be anticipated, caused by symmetry breaking in the excited state. Therefore, **2M** has *C*_1_ symmetry in both the ground and excited states rather than the ideal *C*_3_ symmetry (Figure 5), resulting in a non‐zero dipole moment.

**Table 4:** TD‐DFT calculations on **1M** and **2M** in ethanol solution.

	State	Symmetry	*E* [eV]	*λ* [nm]	*f*	Major (>10 %) contributions^[a]^
**1M**	S_1_	A	3.27	379	1.077	H−1→L (16 %), H→L (72 %)
	S_2_	B	4.12	301	0.011	H−2→L (82 %)
	S_3_	A	4.20	295	0.167	H−9→L (12 %), H−1→L (31 %), H→L+1 (30 %)
**2M** *C*_3_	S_1_	E	3.40	365	1.407	H→L (54 %), H→L+3 (11 %)
	S_2_	E	3.40	365	1.408	H→L+1 (54 %), H→L+4 (11 %)
	S_3_	A	3.66	339	0.000	H−2→L (13 %), H−1→L+1 (13 %), H→L+2 (48 %)
**2M** *C*_1_	S_1_	A	3.40	365	1.407	H→L (56 %), H→L+3 (11 %)
	S_2_	A	3.40	365	1.408	H→L+1 (56 %), H→L+4 (11 %)
	S_3_	A	3.66	339	0.000	H−2→L (16 %), H−1→L+1 (16 %), H→L+2 (48 %)

[a] H: HOMO, L: LUMO.

### Two‐photon absorption

**Figure 6:**
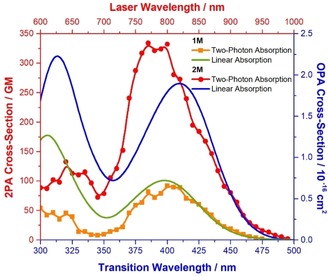
One‐photon absorption spectra of **1M** (green) and **2M** (blue) and two‐photon absorption spectra of **1M** (orange) and **2M** (red) in MeCN.

We measured the two‐photon absorption spectra of both cationic dyes **1M** and **2M** in MeCN via two‐photon excited fluorescence (Figure 6). Although for **1M** the TPA maximum coincides with twice the wavelength of the one‐photon absorption (OPA), the maximum of the TPA spectrum for **2M** is clearly shifted to shorter wavelength (higher energy). This is because TPA from the A symmetry ground state to the A symmetry excited state is allowed whereas it is forbidden for OPA. The energy difference between the TPA energy and the OPA‐allowed E symmetry states thus gives a direct estimate for 3×*V* which is 0.16 eV in reasonable agreement with the DFT computations (3×*V*=0.26 eV, see Table 4). The TPA cross‐section of the dipolar chromophore **1M** is 91 GM in MeCN, which is increased upon 3‐fold branching to 335 GM for **2M**, that is, by a factor of 3.7, and thus there is a small cooperative branching effect for **2M**. This factor is slightly larger (4.2) when estimating the two‐photon cross‐section using the corresponding transition dipole‐moment values ${\left|{\vec{\mu }}_{mi}\right|}^{2}$ and ${\left|{\vec{\mu }}_{fm}\right|}^{2}$ (*σ*_2_≈${\left|{\vec{\mu }}_{mi}\right|}^{2}{\left|{\vec{\mu }}_{fm}\right|}^{2}$ ) where ${\left|{\vec{\mu }}_{mi}\right|}^{2}$ is the transition dipole moment between the ground state and the first one‐photon allowed excited state (for chromophore **1M** is equal to 23.9 D^2^ and for **2M** is 65.0 D^2^), and ${\left|{\vec{\mu }}_{fm}\right|}^{2}$ is the transition dipole moment between the one‐photon allowed state and the first two‐photon allowed excited state (for chromophore **1M** is equal to 29.6 D^2^ and for **2M** is 45.2 D^2^) However, given the general error of the TPA measurement (approx. 10 %) and the expected cooperative behavior (some 10 % at best) we are reluctant to overstress this observation. Despite this conservative assessment, the two‐photon brightness is definitely greatly enhanced by branching because the fluorescence quantum yield also increases with the number of branches. Although dipolar **1M** shows a TPA brightness of 17 GM, the value for octupolar **2M** is enhanced by a factor of about 5 to 87 GM.

### Live‐cell imaging

**Figure 7:**
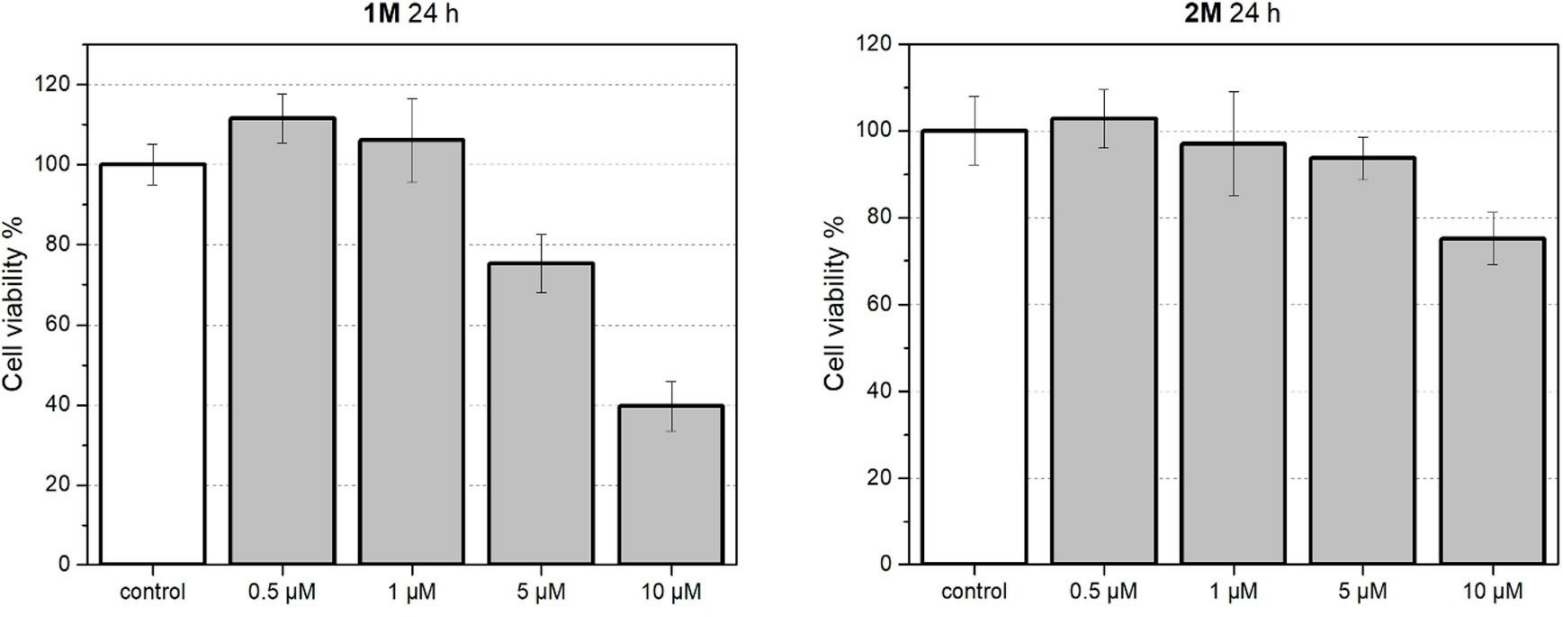
Cell viability of **1M**‐loaded (left) and **2M**‐loaded (right) HeLa cells determined by MTT assay. The cells were incubated with **1M** or **2M** (0, 0.5, 1, 5, 10 μm) in DMEM containing 0.5 % DMSO in a CO_2_ incubator for 24 h. The results are expressed as percentages of the dye‐free controls. All data are presented as a mean with standard deviation (*n=*10).

Before applying the two cationic dyes **1M** and **2M** for live‐cell fluorescence imaging, we tested their influence on the cell viability of HeLa cells. Thus, HeLa cells were treated with serial dilutions of the two compounds, and the cell metabolic activity was studied with a colorimetric (MTT) assay after 24 h (Figure 7). Trace amounts (0.5 %) of DMSO, which do not affect the cell viability, were used to dissolve the compounds in Dulbecco′s modified Eagle′s medium (DMEM) for cell experiments.^34^ Up to a concentration of 1 μm, cell viability is unaffected by either dye, but higher concentrations led to reduced viability. The octupolar chromophore **2M** is less toxic than its dipolar analogue **1M**, for which the cell viability is reduced to 40 % with a staining concentration of 10 μm.

**Figure 8:**
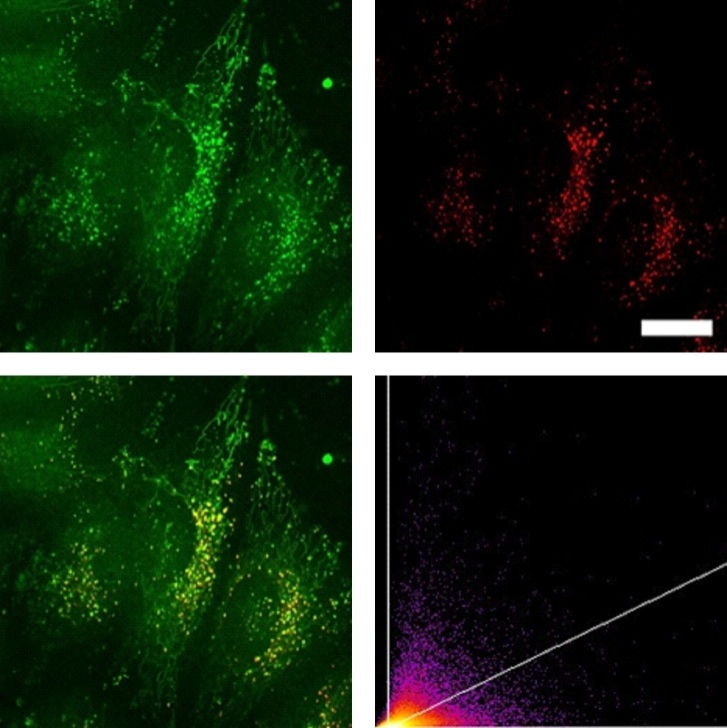
Co‐staining experiment of HeLa cells with **1M** and LysoTracker™ Red. The cells were loaded with **1M** (0.5 μm, 2 h) and LysoTracker™ Red (0.1 μm, 20 min) at 37 °C. Fluorescence images of **1M** (top left, *λ*_ex_=405; *λ*_em_=500–605 nm) and LysoTracker™ Red (top right, *λ*_ex_=561; *λ*_em_=607–786 nm). The merged fluorescence images (bottom left) and the correlation plot of the intensities (bottom right, *R*_r_=0.48) show a modest degree of co‐localization of the dye **1M** in lysosomes. Scale bar: 20 μm.

**Figure 9:**
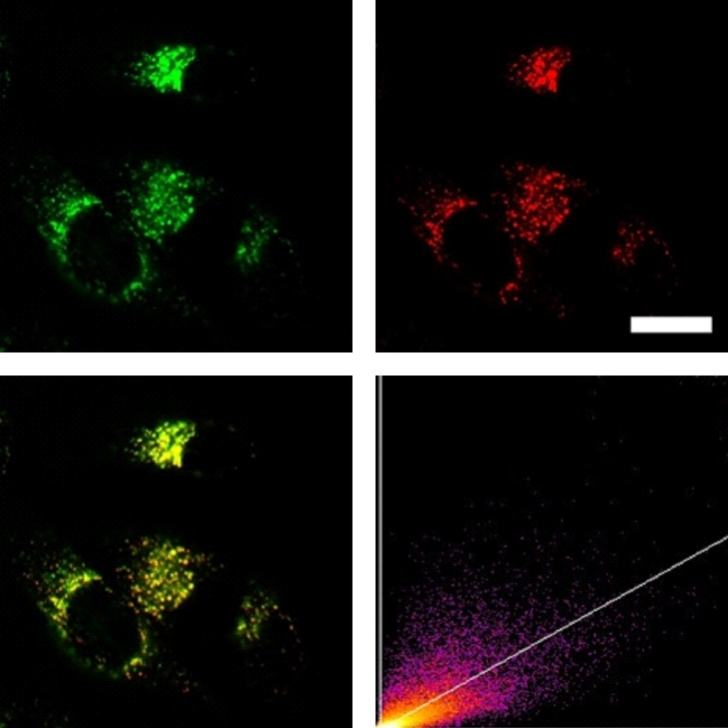
Co‐staining experiment of HeLa cells with **2M** and LysoTracker™ Red. The cells were loaded with **2M** (0.5 μm, 2 h) and LysoTracker™ Red (0.1 μm, 20 min) at 37 °C. Fluorescence images of **2M** (top left, *λ*_ex_=405; *λ*_em_=500–605 nm) and LysoTracker™ Red (top right, *λ*_ex_=561; *λ*_em_=607–786 nm). The merged fluorescence images (bottom left) and the correlation plot of the intensities (bottom right, *R*_r_=0.81) show good co‐localization of the dye **2M** in lysosomes. Scale bar: 20 μm.

**Figure 10:**
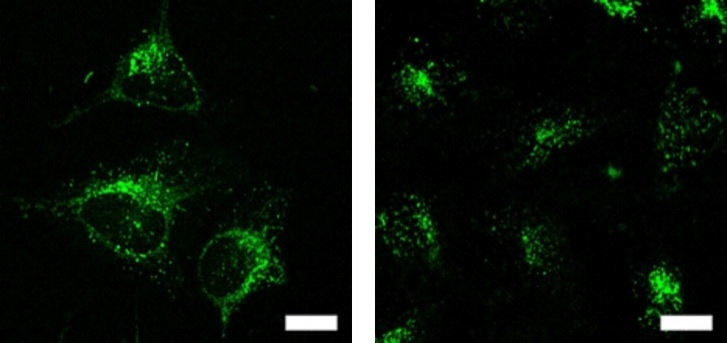
Two‐photon excited fluorescence image of HeLa cells stained with 0.5 μm **1M** (left) or **2M** (right). The TPEF images were recorded with excitation at 800 nm (AOTF 10 %) using an HyD1 detector through a 585/40 bandpass filter and an HCX APO L 40×0.80 W UVI objective. Scale bars: 20 μm.

Having demonstrated that the dyes do not affect the cell viability up to 1 μm after 24 h, we stained HeLa cells with the two dyes **1M** and **2M** (0.5 μm). Using a confocal laser‐scanning fluorescence microscope, we observed cellular uptake of both dyes (Figure 8 and Figure 9). Furthermore, through co‐localization studies with commercially available LysoTracker™ Red, we demonstrate that the octupolar compound **2M** has a very good selectivity for lysosomes with a high Pearson's correlation coefficient (*R*_r_) of 0.81, whereas the dipolar compound **1M** localizes to a lesser extent in lysosomes (*R*_r_=0.48), and is clearly observed elsewhere in the cells. The fiber‐like structures observed in the microscope images may be indicative of some degree of localization in mitochondria. The co‐localization studies with commercially available MitoTracker™ Deep Red further proved that compound **1M** stained both lysosomes and mitochondria (Figure S5 in the Supporting Information). We have recently demonstrated that some multi‐cationic dyes, which were not membrane‐permeable due to electrostatic interaction with negatively charged phospholipids, accumulate on the plasma membrane and are subsequently taken up by cells through the endocytosis process, thus staining lysosomes.8c The observation that octupolar compound **2M** has much better lysosome selectivity than dipolar compound **1M**, is likely related to the lower membrane permeability of **2M** caused by the increased number of cationic groups, its more hydrophilic character, and its larger molecular size. Conversely, compound **1M** is partially membrane permeable and is thus able to stain mitochondria as well. Furthermore, we applied both dyes for TPEF imaging, as shown in Figure 10, and the same staining pattern was observed as in the confocal microscopic imaging using one‐photon excited fluorescence.

## Conclusions

We synthesized two different chromophores, namely dipolar dicationic **1M** with a triphenylamine donor and a dicationic triarylborane acceptor and octupolar hexacationic **2M**, with a triphenylamine core branched by three dicationic triarylborane acceptors. The neutral precursors **1** and **2** show short‐range charge transfer from the dimethylamine donor to the boron acceptor. Therefore, the three subchromophores do not couple with each other because the triphenylamine core is not involved. After methylation, the cationic dyes **1M** and **2M**, behave completely differently. There is a coupling (*V=*0.09 eV) of the three branches observable in the UV/Vis absorption spectrum, because the absorption maxima redshift upon branching. Both systems show a hypsochromic shift with increasing solvent polarity in the absorption spectra, whereas the emission maxima are bathochromically shifted. The cationic dyes **1M** and **2M** show modest cooperative enhancement of the TPA cross‐section (*σ*_2_(**2M**)≈4×*σ*_2_(**1M**)), and an even larger increase (factor of 5) in the two‐photon brightness (*σ*_2_ *Φ*_f_=87 GM) for octupole **2M**. The dyes were applied in TPEF imaging of live cells, and we observed different behaviors of the two systems. The octupolar system **2M** is more biocompatible than the dipolar one **1M**, because the former shows lower cytotoxicity at higher concentrations. Furthermore, the selectivity of the dye **2M** for lysosomes is much better due to the increased number of cationic groups and therefore, more hydrophilic character, and the larger size of the molecule. In summary, the octupolar system **2M** is more suitable for TPEF imaging than the dipolar system **1M**, because the former has a much higher TPA brightness, is less toxic and is more selective for lysosomes. In consideration of the good TPA brightness under excitation at 800 nm, these two dyes are also attractive for in vivo fluorescence imaging which generally requires NIR excitation to obtain deep tissue penetration.

## Conflict of interest

The authors declare no conflict of interest.

## Supporting information

As a service to our authors and readers, this journal provides supporting information supplied by the authors. Such materials are peer reviewed and may be re‐organized for online delivery, but are not copy‐edited or typeset. Technical support issues arising from supporting information (other than missing files) should be addressed to the authors.

Supplementary
