# Supplementary material for: The Effect of Branching on the One‐ and Two‐Photon Absorption, Cell Viability, and Localization of Cationic Triarylborane Chromophores with Dipolar versus Octupolar Charge Distributions for Cellular Imaging
Source: Chemistry. 2019 Sep 17;25(57):13164–75. doi: 10.1002/chem.201902461 (PMC6857003; doi:10.1002/chem.201902461)
Supplement: Supplementary file 1 — Supplementary [file CHEM-25-13164-s001.pdf]

CHEMISTRY
A European Journal

Supporting Information

The Effect of Branching on the One- and Two-Photon Absorption, Cell Viability, and Localization of Cationic Triarylborane Chromophores with Dipolar versus Octupolar Charge Distributions for Cellular Imaging**

Stefanie Griesbeck,[a] Evripidis Michail,[b] Florian Rauch,[a] Hiroaki Ogasawara,[c] Chenguang Wang,[c] Yoshikatsu Sato,[c] Robert M. Edkins,[a, d] Zuolun Zhang,[a, e] Masayasu Taki,[c] Christoph Lambert,*[b] Shigehiro Yamaguchi,*[c] and Todd B. Marder*[a]

chem_201902461_sm_miscellaneous_information.pdf

Contents

Experimental section 2

Linear optical properties 11

TD-DFT calculations 12

Co-localization with MitoTracker™ Deep Red 18

NMR spectra 19

Cartesian coordinates for all DFT-optimized structures 25

References 32

Experimental section

General information. Unless otherwise noted, the following conditions apply. Reactions were performed using standard Schlenk or glovebox (Innovative Technology Inc.) techniques under an atmosphere of argon. Only oven-dried was used. Solvents used for reactions were HPLC grade, dried using an Innovative Technology Inc. Solvent Purification System, and further deoxygenated. Tris(dibenzylideneacetone)-dipalladium(0)[1] and bis[4-(*N*,*N*-dimethylamino)-2,6-xylyl]-2,6-dimethyl-4-(4,4,5,5-tetramethyl-1,3,2-dioxaborlan-2-yl)phenylborane **3**[2] were synthesized according to literature procedures. All other starting materials were purchased from commercial sources and were used without further purification.

Reaction progress was monitored using thin layer chromatography (TLC) plates pre-coated with a layer of silica (Polygram® Sil G/UV254) with fluorescent indicator UV254 from Marchery-Nagel. Automated flash column chromatography was performed using a Biotage® Isolera Four system with silica gel (Biotage SNAP catridge KP-Sil 50g or KP-Sil 100g obtained from Biotage) as the stationary phase and the solvent system indicated. Solvents were generally removed *in vacuo* using a rotary evaporator at a maximum temperature of 50 °C.

^{1}H, ^{13}C{^{1}H} and ^{11}B{^{1}H} solution NMR spectroscopic data were obtained at ambient temperature using a Bruker Avance 500 NMR spectrometer (operating at 500 MHz for ^{1}H, 125 MHz for ^{13}C{^{1}H} and 160 MHz for ^{11}B{^{1}H}). Chemical shifts (δ) were referenced to solvent peaks as follows: ^{1}H NMR spectra were referenced to residual protiated solvent in CD_2Cl_2 (5.32 ppm) or CD_3OD (3.31 ppm); ^{13}C{^{1}H} spectra were referenced to CD_2Cl_2 (53.84 ppm) or CD_3OD (49.00 ppm); and ^{11}B{^{1}H} spectra were referenced to BF_3•Et_2O. The solid-state magic-angle spinning (MAS) NMR spectra were recorded using a Bruker DSX-400 or a Bruker Avance Neo 400 WB spectrometer operating at 128.38 MHz for ^{11}B and a 4 mm rotor (o. d.). Spectra are referenced to external BF_3•Et_2O. Isotropic chemical shifts were estimated by simulating the observed spectrum using the Solid Line Shape Analysis 2.2.4 (SOLA) in Bruker TopSpin. Due to the very small amount of sample of **2M**, a residual boron signal of the boron nitride stator is observable at -20 to 20 ppm.

Elemental analyses were performed on an Elementar vario MICRO cube elemental analyser. As is common for related organo-$BMes_2$ compounds, carbon analysis of **2M** was up to 2.2% below the calculated value, while hydrogen, nitrogen and sulfur analyses were satisfactory. This has been ascribed previously to the formation of boron carbide.[3] High resolution mass spectrometry (MS) was performed with a Thermo Fisher Scientific Exactive Plus Orbitrap MS System with either an Atmospheric Sample Analysis Probe (ASAP) or a heated-electrospray ionization (HESI) probe.

General photophysical measurements. All measurements were performed in standard quartz cuvettes (1 cm x 1 cm cross-section). UV-visible absorption spectra were recorded using an Agilent 8453 diode array UV-visible spectrophotometer. The molar extinction coefficients were calculated from three independently prepared samples in MeCN (**1M**, **2M**) and hexane (**1**, **2**) solution.

The emission spectra were recorded using an Edinburgh Instruments FLSP920 spectrometer equipped with a double monochromator for both excitation and emission, operating in right-angle geometry mode, and all spectra were fully corrected for the spectral response of the instrument. All solutions used in photophysical measurements had a concentration lower than 5×10^{-6} M to minimize inner filter effects during fluorescence measurements.

Fluorescence quantum yield measurements. The fluorescence quantum yields were measured using a calibrated integrating sphere (inner diameter: 150 mm) from Edinburgh Instruments combined with the FLSP920 spectrometer described above. For solution-state measurements, the longest-wavelength absorption maximum of the compound in the respective solvent was chosen as the excitation wavelength, unless stated otherwise.

Lifetime measurements. Fluorescence lifetimes were recorded using the time-correlated single-photon counting (TCSPC) method using an Edinburgh Instruments FLS980 spectrometer equipped with a high speed photomultiplier tube positioned after a single emission monochromator. Measurements were made in right-angle geometry mode, and the emission was collected through a polarizer set to the magic angle. Solutions were excited with a pulsed diode laser at a wavelength of 378 nm (for **1** and **2**) and 419 nm (for **1M** and **2M**) at repetition rates of 10 or 20 MHz, as appropriate. The full-width-at-half-maximum (FWHM) of the pulse from the diode laser was *ca.* 75-90 ps with an instrument response function (IRF) of *ca.* 230 ps FWHM. The IRFs were measured from the scatter of an aqueous suspension of Ludox at the excitation wavelength. Decays were recorded to 10 000 counts in the peak channel with a record length of 8192 channels. The band pass of the emission monochromator and a variable neutral density filter on the excitation side were adjusted to give a signal count rate of <60 kHz. Iterative reconvolution of the IRF with one decay function and non-linear least-squares analysis were used to analyse the data. The quality of all decay fits was judged to be satisfactory, based on the calculated values of the reduced χ^2 and Durbin-Watson parameters and visual inspection of the weighted residuals.

Two-photon absorption measurements. The two-photon absorption cross-section of the compounds was determined by the two-photon induced fluorescence technique. In detail, the fundamental laser source used is an amplified Ti: sapphire laser (Solstice, Spectra Physics) operating at 1 KHz repetition rate with 100 fs pulses at 800 nm. 70% of the of the available energy seeds a computer-controlled optical parametric amplifier (TOPAS-C, Light

Conversion), which produces pulses in the range of 290 - 2600 nm. Excitation of the samples was achieved using a protected silver parabolic mirror, using vertically polarized light with the excitation energy varying in the 0.2 – 3 µJ range. The latter conditions were established by using a series of three thin broadband polarizers and a mechanical rotational mount. Maintenance of identical excitation conditions for both reference and samples was achieved using a high-precision motorized rotational stage to ensure that the unknown compounds and the secondary reference standard are subjected to the same excitation conditions. Coumarin 540A in CCl_4 and Styryl 9M in $CHCl_3$ were used as reference compounds.[4] The emitted fluorescence signal was detected at 90° with respect to the excitation beam, and recorded using a compact CCD spectrometer. Two-photon excitation was verified by log-log plots of fluorescence intensities vs. excitation power at various wavelengths, all giving slopes of 2.

Transition Dipole Moment Calculation. The squares of the transition dipole moment $|\mu_{\mathrm{mi}}|^2$ and $|\mu_{\mathrm{fm}}|^2$ were calculated from the following equations:

$$|\mu_{\mathrm{mi}}|^2 = \frac{3hc\varepsilon_0 \ln(10)}{2000\,(\pi^2)\,N_A} \frac{9n}{(n^2+2)^2} \int \frac{\varepsilon_{\mathrm{ge}}(\tilde{k})}{\tilde{k}} d\tilde{k}$$

where h is the Planck´s constant, c is the speed of light, ε_0 is the vacuum permittivity, n is the refractive index of the medium, N_A is Avogadro´s number and $\varepsilon_{\mathrm{ge}}(\tilde{k})$ the extinction coefficient at the wavenumber $\tilde{k}$; The integral is over the $S_0 \rightarrow S_1$ absorption band, and

$$|\mu_{\mathrm{fm}}|^2 = \frac{5h^2n^2c^2}{32\pi^4L^4}\left[\frac{1}{v_{max}^2}\right]\frac{[(v_{\mathrm{mi}} - v_{max})^2 + \Gamma(v)^2]}{|\mu_{\mathrm{mi}}|^2}\left(\frac{1}{G_{\mathrm{vib.}}}\right)[\sigma_{\mathrm{max}}\,(v)]$$

where L is the local field factor, v_{max} is the transition frequency of the 2PA cross-section maximum $\sigma_{\mathrm{max}}\,(v)$, the bracket $(v_{\mathrm{mi}} - v_{max})$ is the detuning frequency and can be calculated from the absorption peaks in the one- and two- photon spectra. Finally, the term $G_{\mathrm{vib.}}$ is a Gaussian line shape function that is centered at the energy maximum for the two-photon allowed state in order to include the vibrational transitions. The magnitudes of the 2PA cross-sections σ_2 were estimated using a three-level model. Thus, the product of the square of the transition dipole moments are analogous to the 2PA cross-section: ($\sigma_2 \propto |\vec{\mu}_{\mathrm{mi}}|^2\,|\vec{\mu}_{\mathrm{fm}}|^2$).

Theoretical studies. All calculations (DFT and TD-DFT) were carried out with the Gaussian 09 (Rev. D/E.01)[5] program package and were performed on a parallel cluster system. GaussView 6.0.9 was used to visualize the results, to measure calculated structural parameters, and to plot orbital surfaces (isovalue: ±0.03 [e a_0^{-3}]$^{1/2}$). The ground-state geometries were optimized using the B3LYP functional[6] in combination with the 6-31G(d) basis set.[7] The optimized geometries were confirmed to be local minima by performing frequency calculations and obtaining only positive (real) frequencies. Where optimized structures of a higher symmetry (>C_1) were determined as local minima by frequency

calculation, the symmetry was included in the subsequent calculations as well. Stability analysis showed the wavefunction to be stable in each case. Based on these optimized structures, the lowest-energy gas-phase vertical transitions were calculated (singlets, 25 states) by TD-DFT, using the Coulomb-attenuated functional CAM-B3LYP[8] in combination with the 6-31G(d) basis set.[6a, 6b] The CAM-B3LYP functional has been shown to be effective for the ICT systems, hence its selection here.[9] The polarizable continuum model (PCM) was used to include solvent effects in the TD-DFT calculations.[10] Natural transition orbitals were calculated for the lowest three energy transitions in each case using Multiwfn.[11] The ultrafine integration grid was used throughout.

Cell culture. HeLa cells (RIKEN Cell Bank, Japan) were cultured in Dulbecco's modified Eagle's medium (DMEM, Wako) containing 10% fetal bovine serum (FBS, Biosera) and 1% Antibiotic-Antimycotic (AA, Wako) at 37 °C in a 5% CO_2/95% air incubator. Cells (5 ×10^4) were seeded in poly-lysine coated glass-bottom dishes three days before imaging.

Co-staining experiments with LysoTracker™ Red. The incubation medium was removed from the cells, and the cells were further incubated with 0.5 μM **1M** or **2M** in DMEM containing 0.5% DMSO for 2 h in a CO_2 incubator. Then the cells were rinsed with DMEM three times and the dish was filled with 2.0 mL of DMEM containing 0.1 μM LysoTracker™ Red (Invitrogen) and further incubated for 20 min in a CO_2 incubator. Fluorescence images were obtained with a confocal microscope (TCS SP8 STED 3X; Leica), including an inverted DMI6000 CS microscope equipped with a laser diode (405 nm), a tunable (470 – 670 nm) pulsed white light laser (WLL; repetition rate of 78 MHz) for excitation. For confocal imaging, a HyD detector and 100 × oil-immersion objective (NA 1.4) were used. The dyes were excited with the 405 nm diode laser (**1M**, **2M**), and the 561 nm wavelength of the WLL (LysoTracker™ Red). The fluorescence signals were collected between 500 and 605 nm (**1M**, **2M**) and 607 and 786 nm (LysoTracker™ Red) with a time gating interval of 0.1 − 12 ns. Each image was recorded with a line average of 4. To avoid crosstalk among the channels, the emission signals were collected independently in the sequential scanning mode. Images were processed with Fiji software.

Co-staining experiments with MitoTracker™ Deep Red. The incubation medium was removed from the cells, and the cells were further incubated with 0.05 μM MitoTracker™ Deep Red in DMEM containing 0.5% DMSO for 20 min in a CO_2 incubator. Then the cells were rinsed with DMEM three times and the dish was filled with 2.0 mL of DMEM containing 0.5 μM **1M** and 0.5% DMSO and further incubated for 2 h in a CO_2 incubator. Fluorescence images were obtained with a confocal microscope (TCS SP8 STED 3X; Leica), including an inverted DMI6000 CS microscope equipped with a laser diode (405 nm), a tunable (470 – 670 nm) pulsed white light laser (WLL; repetition rate of 78 MHz) for excitation. For confocal imaging, a

HyD detector and 63 × oil-immersion objective were used. The dyes were excited with the 405 nm diode laser (**1M**), and the 633 nm wavelength of the WLL (MitoTracker™ Deep Red). The fluorescence signals were collected between 500 and 605 nm (**1M**) and 645 and 786 nm (MitoTracker™ Deep Red) with a time gating interval of 0.1 − 12 ns. Each image was recorded with a line average of 4. To avoid crosstalk among the channels, the emission signals were collected independently in the sequential scanning mode. Images were processed with Fiji software.

Cytotoxicity evaluation. HeLa cells were seeded into a flat-bottomed 96-well plate (1 $\times 10^4$ cells/well) and incubated in DMEM at 37 °C in a 5% CO_2/95% air incubator for 24 h. The medium was then replaced with culture medium (100 μL/well) containing various concentrations of **1M** or **2M** (0, 0.5, 1, 5, and 10 μM) in DMEM (0.5% DMSO). After the cells were incubated for 24h at 37 °C, MTT reagent (10 μL/well, 0.5 mg/mL) in PBS was added to each well, and the plates were incubated for another 4 h in a CO_2 incubator. The medium was then removed, the formazan crystals were solubilized in DMSO (100 μL/well) for 10 min at room temperature, and the absorbance of each well was measured using a SpectraMax i3 (Molecular Devices) with an excitation at 535 nm.

TPA imaging. The incubation medium was removed from the cells, and the cells were further incubated with 0.5 μM **1M** or **2M** in DMEM in a CO_2 incubator for 2 h. Then the cells were rinsed with DMEM three times and the dish was filled with 15 mL of DMEM containing 20 mM HEPES (pH = 7.4). Imaging experiments were conducted using a Leica SP8-MP system (Leica Microsystems), equipped with a tunable (680 - 1300 nm) laser for two-photon excitation. For TPA imaging, a HyD detector with a 585/40 bandpass filter and a Lens HCX APO L 40 × 0.80 W UVI objective were used. The dyes were excited with the 800 nm wavelength of the laser and fluorescence signals were collected through a 585/40 band pass filter.

Synthesis

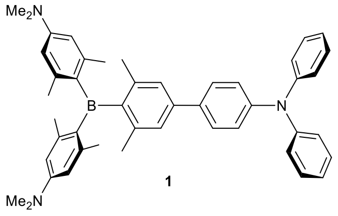

1

4-[4-[Bis[4-(dimethylamino)-2,6-xylyl]boryl]-3,5-xylyl]-phenyl-*N*,*N*-diphenylamine (1)

Compound **3** (537 mg, 0.93 mmol, 1.1 eq.), 4-bromo-*N*,*N*-diphenylaniline (273 mg, 0.85 mmol, 1 eq.), KOH (157 mg, 2.79 mmol, 3 eq.) and SPhos (38 mg, 0.09 mmol, 10 mol%) were dissolved in a degassed mixture of toluene (10 mL) and H_2O (5 mL). The reaction mixture was degassed and $Pd_2(dba)_3$ (26 mg, 0.03 mmol, 3 mol%) was added. The reaction was heated to 85 °C and stirred for 20 h. Reaction monitoring by TLC (hexane:EtOAc 8:1) indicated that the starting material was fully consumed. Therefore, the phases were separated and the aqueous solution was extracted with hexane (3 x 10 mL). The combined organic layers were concentrated under reduced pressure and purified by column chromatography (silica gel, hexane:EtOAc 12:1). The yellow solid was dissolved in Et_2O and crystallized by adding MeOH to afford **1** as a yellow solid (441 mg, 79%).

^{1}H NMR (500 MHz, CD_2Cl_2): δ = 7.56 – 7.54 (m, 2H), 7.28 – 7.25 (m, 4H), 7.15 (s, 2H), 7.12 – 7.09 (m, 6H), 7.05 – 7.01 (m, 2H), 6.32 (br s, 2H), 6.31 (br s, 2H), 2.95 (s, 12H), 2.08 (s, 6H), 2.02 (s, 6H), 1.97 (s, 6H) ppm.

^{13}C{^{1}H} NMR (125 MHz, CD_2Cl_2): δ = 151.7, 148.4, 148.2, 147.4, 143.2, 142.9, 141.1, 140.2, 136.2, 135.6, 129.6, 127.7, 125.6, 124.7, 124.4, 123.2, 111.9, 111.8, 40.2, 24.0, 23.8, 23.1 ppm. Additional carbon peaks are due to hindered rotation around B–C bonds.

^{11}B{^{1}H} NMR (160 MHz, CD_2Cl_2): δ = 74 ppm.

HRMS (APCI$^+$): *m/z* found: $[M+H]^+$ 656.4160; calc. for $[C_{46}H_{51}BN_3]^+$ 656.4171 ($|\Delta|$ = 1.68 ppm).

Elem. Anal. Calc. (%) for $C_{46}H_{50}BN_3$: C 84.26, H 7.69, N 6.41; found: C 84.39, H 7.67, N 6.35.

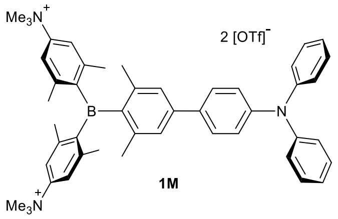

4-[4-[Bis[4-(trimethylammonio)-2,6-xylyl]boryl]-3,5-xylyl]-phenyl-*N*,*N*-diphenylamine ditriflate (1M)

The neutral donor-acceptor compound **1** (100 mg, 0.15 mmol, 1 eq.) was dissolved in dry CH_2Cl_2 (15 mL) and then methyl triflate (86 µL, 0.76 mmol, 5 eq.) was added. The reaction mixture was stirred for 20 h at r.t.. The yellow solid which precipitated upon additional Et_2O (20 mL) was collected by filtration and washed with Et_2O to afford **1M** (152 mg, 97%).

^{1}H NMR (500 MHz, CD_2Cl_2): δ = 7.57 – 7.56 (m, 6H), 7.30 – 7.27 (m, 6H), 7.09 – 7.04 (m, 8H), 3.66 (s, 18H), 2.24 (s, 6H), 2.16 (s, 6H), 2.08 (s, 6H) ppm.

^{13}C{^{1}H} NMR (125 MHz, CD_2Cl_2): δ = 149.6, 149.2, 149.0, 144.9, 144.5, 144.4, 142.8, 135.0, 130.5, 128.7, 127.3, 125.8, 124.4, 124.4 121.8 (q, $J_{C\text{-}F}$ = 318 Hz), 120.1, 120.0, 57.5, 23.6, 23.4 ppm.

Solid-State ^{11}B{^{1}H} NMR (128 MHz): Isotropic chemical shift δ_{iso} = 76.0 and 77.6 ppm, quadrupole coupling constant C_Q = 4.76 and 4.78 MHz, quadrupolar asymmetry parameter η_{Quad} = 0.0 and 0.0. Since no further side products are observable via NMR spectroscopy in solution, it can be assumed that the two signals correspond to two isomers, which exist in the solid state. An integration of the signals gives a ratio of 1:1.

HRMS (ESI$^+$): *m/z* found: [M-OTf]$^+$ 834.4074; calc. for $[C_{49}H_{56}BN_3SO_3F_3]^+$ 834.4082 (|Δ| = 0.96 ppm).

Elem. Anal. Calc. (%) for $C_{50}H_{56}BN_3F_6O_6S_2$: C 61.03, H 5.74, N 4.27, S 6.52; found: C 60.74, H 5.74, N 4.50, S 6.36.

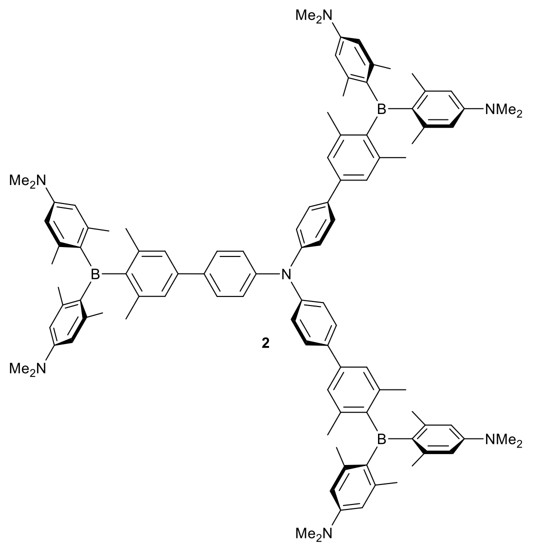

Tris[4-[4-[bis[4-(dimethylamino)-2,6-xylyl]boryl]-3,5-xylyl]-phenyl]amine (2)

Compound **3** (500 mg, 0.93 mmol, 3.1 eq.), 4,4′,4″-tribromotriphenylamine (144 mg, 0.30 mmol, 1 eq.), KOH (152 mg, 2.70 mmol, 9 eq.) and SPhos (37 mg, 0.09 mmol, 30 mol%) were dissolved in a degassed mixture of toluene (10 mL) and H_2O (5 mL). The reaction mixture was degassed and $Pd_2(dba)_3$ (25 mg, 0.02 mmol, 8 mol%) was added. The reaction was heated to 85 °C and stirred for 20 h. Reaction monitoring by TLC (hexane:EtOAc 3:1) indicated that the starting material was consumed. Therefore, the phases were separated, and the aqueous solution was extracted with hexane (3 x 10 mL). The combined organic layers were concentrated under reduced pressure, and the residue was purified by column chromatography (silica gel, hexane:EtOAc 3:1). The yellow solid was dissolved in Et_2O and crystallized by adding MeOH to afford **2** as a yellow solid (389 mg, 88%).

^{1}H NMR (500 MHz, CD_2Cl_2): δ = 7.60 – 7.58 (m, 6H), 7.21 – 7.19 (m, 6H), 7.18 (s, 6H), 6.33 (br s, 6H), 6.32 (br s, 6H), 2.95 (s, 36H), 2.09 (s, 18H), 2.02 (s, 18H), 1.98 (s, 18H) ppm.

^{13}C{^{1}H} NMR (125 MHz, CD_2Cl_2): δ = 151.6, 148.5, 147.1, 143.2, 142.9, 141.1, 140.2, 136.2, 135.8, 127.8, 125.6, 124.7, 111.9, 111.8, 40.2, 24.0, 23.8, 23.1 ppm. Additional carbon peaks are due to hindered rotation around B–C bonds.

Solid-State ^{11}B{^{1}H} NMR (128 MHz): Isotropic chemical shift δ_{iso} = 73.2 ppm, quadrupole coupling constant C_Q = 4.53 MHz, quadrupolar asymmetry parameter η_{Quad} = 0.0.

HRMS (APCI$^+$): *m/z* found: $[M+H]^+$ 1476.9989; calc. for $[C_{102}H_{121}B_3N_7]^+$ 1476.9969 (|Δ| = 0.40 ppm).

Elem. Anal. Calc. (%) for $C_{102}H_{120}B_3N_7$: C 82.97, H 8.19, N 6.64; found: C 82.70, H 8.25, N 6.47.

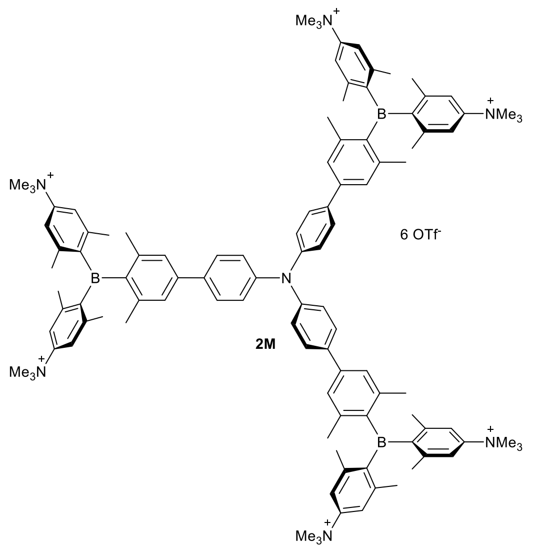

Tris[4-[4-[bis[4-(trimethylammonio)-2,6-xylyl]boryl]-3,5-xylyl]-phenyl]amine hexatriflate (2M)

The neutral trigonal donor-acceptor compound **2** (70 mg, 0.05 mmol, 1 eq.) was dissolved in dry CH_2Cl_2 (4.7 mL) and Et_2O (1.6 mL) and then methyl triflate (80 μL, 0.71 mmol, 15 eq.) was added. The reaction mixture was stirred for 20 h at r.t. The yellow solid which precipitated upon addition of Et_2O (20 mL) was collected by filtration and washed with Et_2O to afford **2M** as a yellow solid (107 mg, 92%).

^{1}H NMR (500 MHz, CD_2Cl_2): δ = 7.67 – 7.65 (m, 6H), 7.57 (br s, 6H), 7.57 (br s, 6H), 7.34 (s, 6H), 7.23 – 7.21 (m, 6H), 3.66 (s, 54H), 2.25 (s, 18H), 2.16 (s, 18H), 2.10 (s, 18H) ppm.

^{13}C{^{1}H} NMR (125 MHz, CD_2Cl_2): δ = 149.6, 149.5, 148.6, 144.9, 144.8, 144.5, 144.4, 142.9, 136.1, 128.9, 127.4, 125.6, 121.8 (q, J = 316 Hz), 120.1, 120.1, 57.5, 23.6, 23.6, 23.4 ppm. Additional carbon peaks are due to hindered rotation around B–C bonds.

Solid-State ^{11}B{^{1}H} NMR (128 MHz): Isotropic chemical shift δ_{iso} = 77.6 ppm, quadrupole coupling constant C_Q = 4.79 MHz, quadrupolar asymmetry parameter η_{Quad} = 0.1.

HRMS (ESI$^+$): *m/z* found: $[M\text{-}6OTf]^{6+}$ 261.0216; calc. for $[C_{108}H_{138}B_3N_7]^+$ 261.0210 (|Δ| = 2.30 ppm).

Elem. Anal. Calc. (%) for $C_{114}H_{138}B_3N_7F_{18}O_{18}S_6$: C 55.63, H 5.65, N 3.98, S 7.82; found: C 54.45, H 5.89, N 3.99, S 7.53.

Linear optical properties

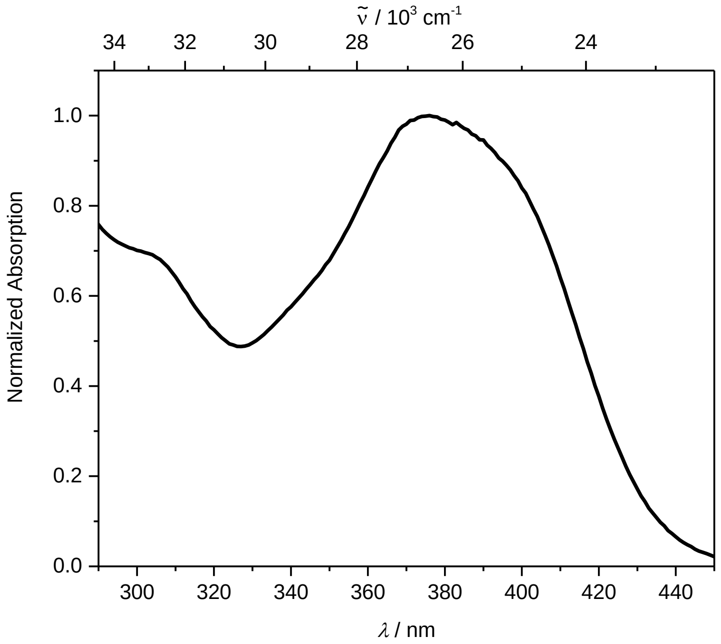

Figure S1. Absorption spectrum of **1** in MeCN.

TD-DFT calculations

Table S1. Lowest energy singlet electronic transitions of **1** in the gas phase. H = HOMO; L = LUMO.

State	Symmetry	*E* (eV)	λ (nm)	*f*	Major (> 10%) contributions
S_1	A	3.56	349	0.327	H-1 → L (86%)
S_2	A	3.75	331	0.708	H-3 → L (13%), H-2 → L (41%), H → L (32%)
S_3	A	4.15	299	0.320	H-2 → L (42%), H → L (23%), H → L+1 (24%)
S_4	A	4.44	279	0.018	H → L+2 (84%)
S_5	A	4.50	276	0.138	H-3 → L (40%), H → L+1 (35%)
S_6	A	4.55	272	0.267	H → L+3 (87%)
S_7	A	4.60	270	0.004	H-4 → L (60%)
S_8	A	4.63	268	0.025	H-5 → L (70%)
S_9	A	4.66	266	0.000	H-6 → L (51%), H-1 → L+8 (11%)
S_{10}	A	4.92	252	0.020	H → L+4 (50%), H → L+6 (14%)

Table S2. Lowest energy singlet electronic transitions of **1M** in the gas phase. H = HOMO; L = LUMO.

State	Symmetry	*E* (eV)	λ (nm)	*f*	Major (> 10%) contributions
S_1	A	2.33	533	0.616	H → L (89%)
S_2	B	3.57	347	0.004	H → L+1 (94%)
S_3	A	3.60	345	0.417	H-7 → L (15%), H-3 → L (20%), H-1 → L (20%), H → L (11%), H → L+2 (14%)
S_4	A	3.69	336	0.228	H-7 → L (10%), H-3 →L (10%), H → L+2 (41%), H → L+4 (18%)
S_5	B	3.78	328	0.012	H-6 → L (89%)
S_6	B	4.02	308	0.001	H → L+3 (96%)
S_7	A	4.18	296	0.060	H → L+2 (37%), H → L+4 (54%)
S_8	B	4.26	291	0.001	H-5 → L (33%), H-2 → L (28%), H → L+11 (17%)
S_9	A	4.37	284	0.000	H-3 → L (22%), H-1 → L (65%)
S_{10}	B	4.47	277	0.012	H-2 → L (41%), H → L+7 (19%), H → L+11 (30%)

Table S3. Lowest energy singlet electronic transitions of **1M** in EtOH solution. H = HOMO; L = LUMO.

State	Symmetry	*E* (eV)	λ (nm)	*f*	Major (> 10%) contributions
S_1	A	3.27	379	1.077	H-1 → L (16%), H → L (72%)
S_2	B	4.12	301	0.011	H-2 → L (82%)
S_3	A	4.20	295	0.167	H-9 → L (12%), H-1 → L (31%), H → L+1 (30%)
S_4	B	4.33	287	0.142	H-5 → L (75%)
S_5	B	4.41	281	0.053	H → L+4 (79%)
S_6	A	4.56	272	0.260	H-9 → L (14%), H → L (19%), H → L+1 (33%)
S_7	B	4.64	267	0.198	H → L+6 (81%)
S_8	A	4.70	264	0.045	H-9 → L (10%), H-6 → L (66%)
S_9	B	4.76	261	0.036	H-8 → L (61%)
S_{10}	A	4.89	253	0.020	H-11 → L (28%), H-9 → L (37%), H-1 → L (18%)

Table S4. Lowest energy singlet electronic transitions of **2** in the gas phase with C_3 symmetry. H = HOMO; L = LUMO.

State	Symmetry	*E* (eV)	*λ* (nm)	*f*	Major (> 10%) contributions
S_1	E	3.56	348	0.161	H-3 → L (27%), H-2 → L+1 (13%), H-2 → L+2 (25%), H-1 → L (13%)
S_2	E	3.56	348	0.161	H-3 → L+1 (27%), H-2 → L (13%), H-1 → L+1 (13%), H-1 → L+2 (25%)
S_3	A	3.56	348	0.611	H-3 → L+2 (29%), H-2 → L (28%), H-1 → L+1 (28%)
S_4	E	3.67	338	1.351	H-4 → L+2 (12%), H → L (30%)
S_5	E	3.67	338	1.351	H-5 → L+2 (12%), H → L+1 (30%)
S_6	A	3.82	325	0.000	H-6 → L+2 (18%), H-5 → L+1 (22%), H-4 → L (22%), H → L+2 (12%)
S_7	E	4.04	307	0.158	H-6 → L+1 (18%), H-5 → L+2 (13%), H → L+1 (16%), H → L+4 (17%)
S_8	E	4.04	307	0.158	H-6 → L (18%), H-4 → L+2 (13%), H → L (16%), H → L+3 (17%)
S_9	A	4.29	289	0.000	H-8 → L (15%), H-7 → L+1 (15%), H-6 → L+2 (12%), H → L+2 (31%),
S_{10}	A	4.35	285	0.013	H → L+5 (84%)

Table S5. Lowest energy singlet electronic transitions of **2** in the gas phase with C_1 symmetry. H = HOMO; L = LUMO.

State	Symmetry	*E* (eV)	*λ* (nm)	*f*	Major (> 10%) contributions
S_1	A	3.56	348	0.161	H-3 → L (12%), H-2 → L (11%), H-1 → L+1 (19%), H-1 → L+2 (26%)
S_2	A	3.56	348	0.161	H-3 → L (21%), H-3 → L+1 (15%), H-2 → L+1 (14%), H-2 → L+2 (28%)
S_3	A	3.56	348	0.611	H-3 → L+2 (27%), H-2 → L (20%), H-1 → L+1 (19%)
S_4	A	3.67	338	1.351	H → L (34%)
S_5	A	3.67	338	1.351	H → L+1 (34%)
S_6	A	3.82	325	0.000	H-6 → L+2 (18%), H-5 → L (12%), H-5 → L+1 (10%), H-4 → L (10%), H-4 → L+1 (12%), H → L+2 (12%)
S_7	A	4.04	307	0.158	H-6 → L (18%), H-4 → L+2 (10%), H → L (17%), H → L+3 (16%)
S_8	A	4.04	307	0.158	H-6 → L+1 (18%), H-5 → L+2 (10%), H → L+1 (17%), H → L+4 (16%)
S_9	A	4.29	289	0.000	H-8 → L+1 (14%), H-7 → L (14%), H-6 → L+2 (12%), H → L+2 (31%),
S_{10}	A	4.35	285	0.013	H → L+5 (84%)

Table S6. Lowest energy singlet electronic transitions of **2M** in the gas phase with C_3 symmetry. H = HOMO; L = LUMO.

State	Symmetry	*E* (eV)	*λ* (nm)	*f*	Major (> 10%) contributions
S_1	E	3.11	398	1.054	H → L (66%)
S_2	E	3.11	398	1.054	H → L+1 (66%)
S_3	A	3.32	373	0.000	H → L+2 (70%)
S_4	E	3.96	313	0.007	H-8 → L (11%), H-5 → L (10%), H-1 → L+2 (11%)
S_5	E	3.96	313	0.007	H-8 → L+1 (11%), H-5 → L+1 (10%), H-2 → L+2 (11%)
S_6	A	3.96	313	0.028	H-5 → L+2 (28%), H-4 → L+1 (22%), H-3 → L (22%)
S_7	E	3.96	313	0.002	H-5 → L (12%), H-4 → L+2 (12%)
S_8	E	3.96	313	0.002	H-5 → L+1 (12%), H-3 → L+2 (12%)
S_9	A	3.99	311	0.000	H-8 → L+2 (16%), H-2 → L+1 (18%), H-1 → L (18%), H → L+2 (27%)
S_{10}	E	4.21	294	0.622	H → L+1 (14%), H → L+6 (36%), H → L+7 (14%)

Table S7. Lowest energy singlet electronic transitions of **2M** in EtOH solution with C_3 symmetry. H = HOMO; L = LUMO.

State	Symmetry	*E* (eV)	λ (nm)	*f*	Major (> 10%) contributions
S_1	E	3.40	365	1.407	H → L (54%), H → L+3 (11%)
S_2	E	3.40	365	1.408	H → L+1 (54%), H → L+4 (11%)
S_3	A	3.66	339	0.000	H-2 → L (13%), H-1 → L+1 (13%), H → L+2 (48%),
S_4	E	4.12	301	0.014	H-6 → L (14%), H-1 → L+2 (10%), H → L+3 (17%)
S_5	E	4.12	301	0.013	H-6 → L+1 (14%), H-2 → L+2 (10%), H → L+4 (17%)
S_6	E	4.15	298	0.006	H-5 → L+2 (10%), H-4 → L (26%), H-4 → L+2 (13%), H-3 → L (12%), H-3 → L+1 (12%)
S_7	E	4.15	298	0.017	H-4 → L+1 (14%), H-3 → L+1 (26%), H-3 → L+2 (22%)
S_8	E	4.15	298	0.017	H-5 → L (23%), H-5 → L+1 (18%), H-5 → L+2 (12%), H-4 → L+2 (11%)
S_9	E	4.30	288	0.012	H-10 → L+2 (12%), H-9 → L+1 (23%)
S_{10}	E	4.30	288	0.018	H-11 → L+1 (9%)

Table S8. Lowest energy singlet electronic transitions of **2M** in EtOH solution with C_1 symmetry. H = HOMO; L = LUMO.

State	Symmetry	*E* (eV)	λ (nm)	*f*	Major (> 10%) contributions
S_1	A	3.40	365	1.407	H → L (56%), H → L+3 (11%)
S_2	A	3.40	365	1.408	H → L+1 (56%), H → L+4 (11%)
S_3	A	3.66	339	0.000	H-2 → L (16%), H-1 → L+1 (16%), H → L+2 (48%),
S_4	A	4.12	301	0.014	H-6 → L (18%), H-2 → L+2 (14%), H → L+3 (23%)
S_5	A	4.12	301	0.014	H-6 → L+1 (18%), H-1 → L+2 (14%), H → L+4 (23%)
S_6	A	4.15	299	0.003	H-5 → L+2 (15%), H-4 → L (11%), H-3 → L+1 (37%)
S_7	A	4.15	299	0.017	H-5 → L (10%), H-5 → L+1 (11%), H-4 → L (22%), H-3 → L+2 (14%)
S_8	A	4.15	299	0.020	H-5 → L (26%), H-4 → L+1 (17%), H-4 → L+2 (23%)
S_9	A	4.30	288	0.013	H-10 → L+2 (10%), H-9 → L+1 (12%)
S_{10}	A	4.30	288	0.012	H-9 → L+1 (10%)

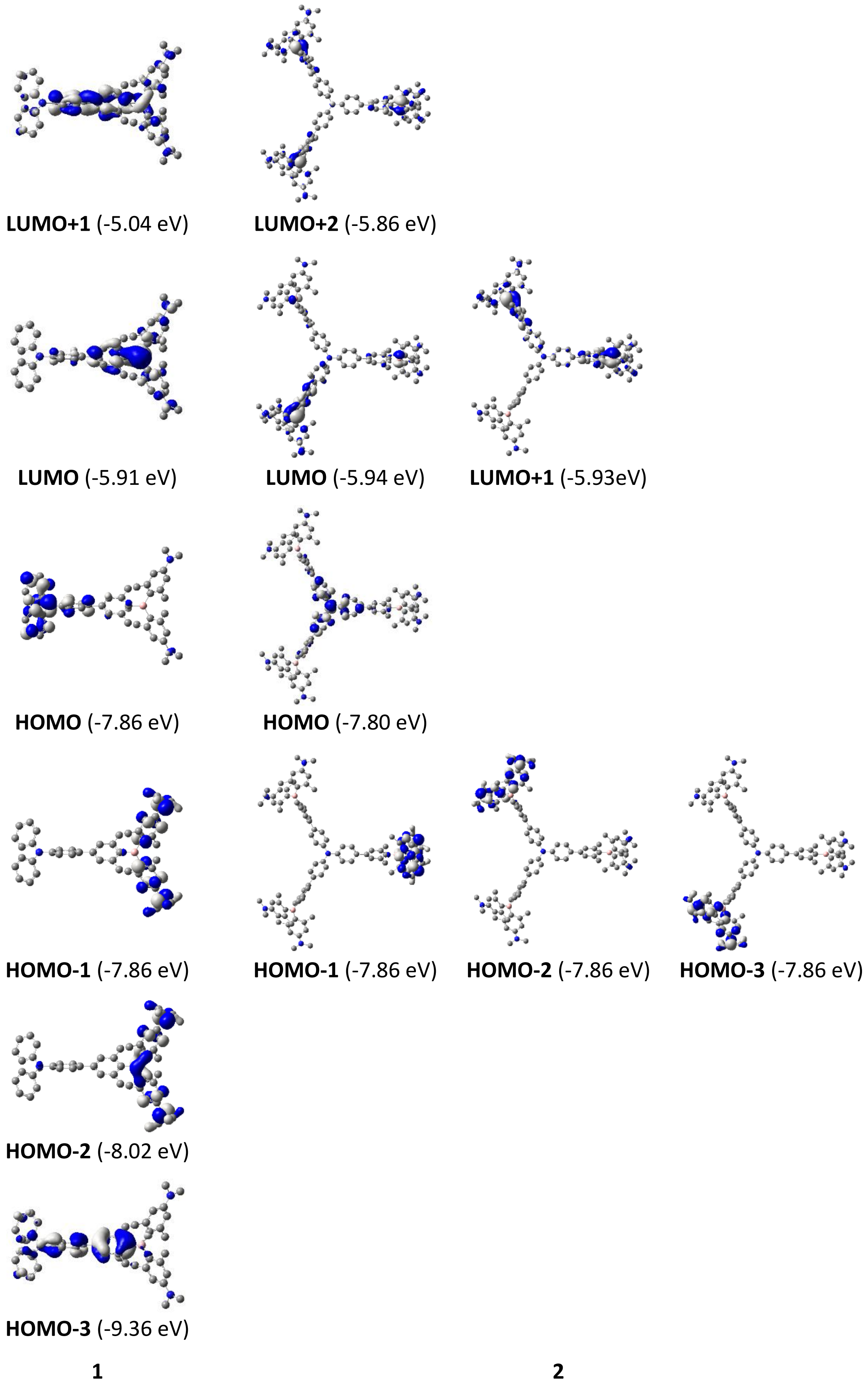

Figure S2. DFT (CAM-B3LYP/6-31 G(d))-calculated frontier orbitals for **1** and **2**. Hydrogen atoms are omitted for clarity. Surface isovalue: ± 0.03 [e a_0^{-3}]$^{1/2}$. LUMO and LUMO+1, as well as HOMO-1, HOMO-2 and HOMO-3 of **2** are isoenergetic.

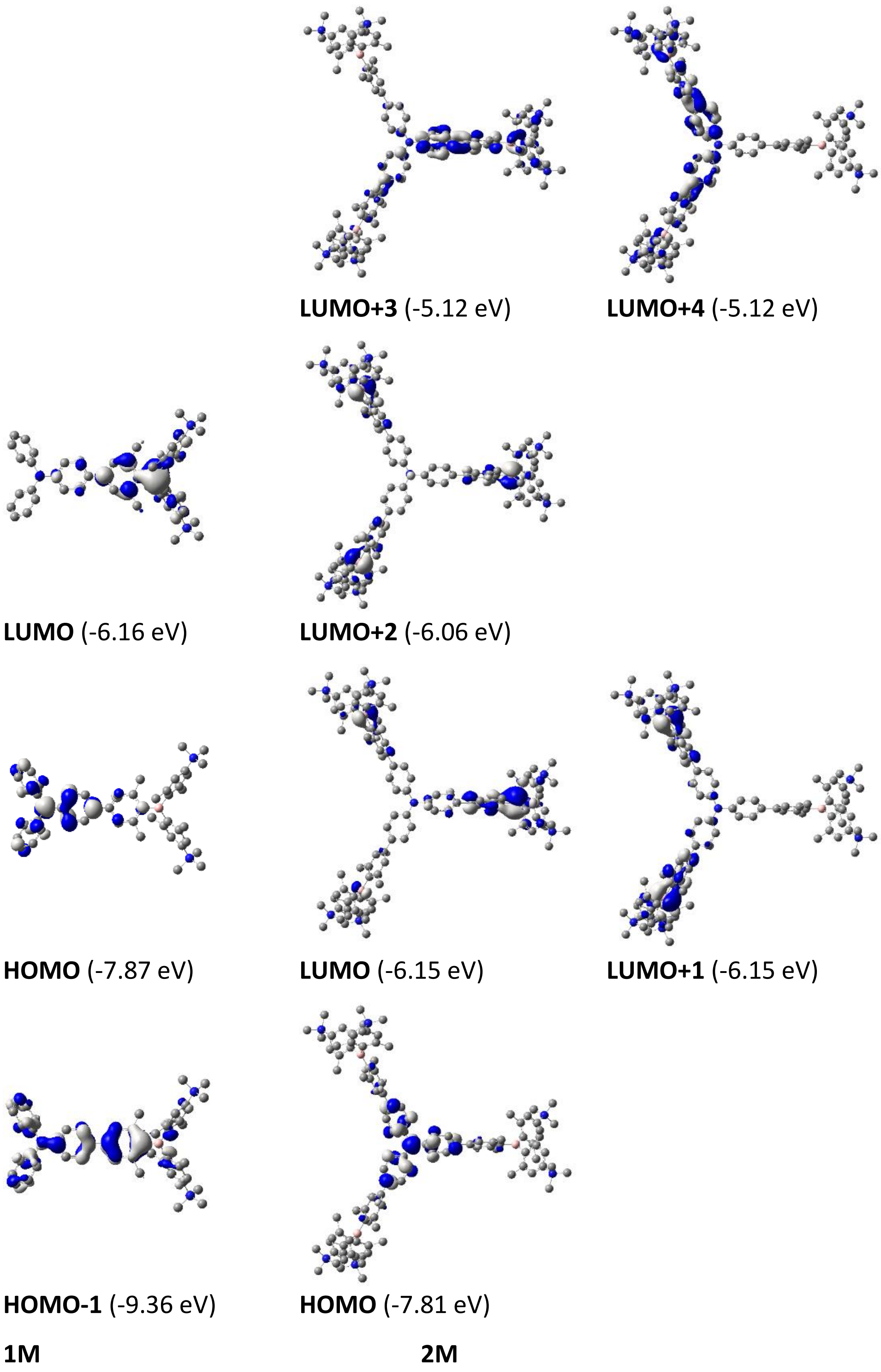

Figure S3. DFT (CAM-B3LYP/6-31 G(d))-calculated frontier orbitals for **1M** and **2M**. Hydrogen atoms are omitted for clarity. Surface isovalue: ± 0.03 [e a_0^{-3}]$^{1/2}$. LUMO and LUMO+1, as well as LUMO+3 and LUMO+4 of **2M** are isoenergetic.

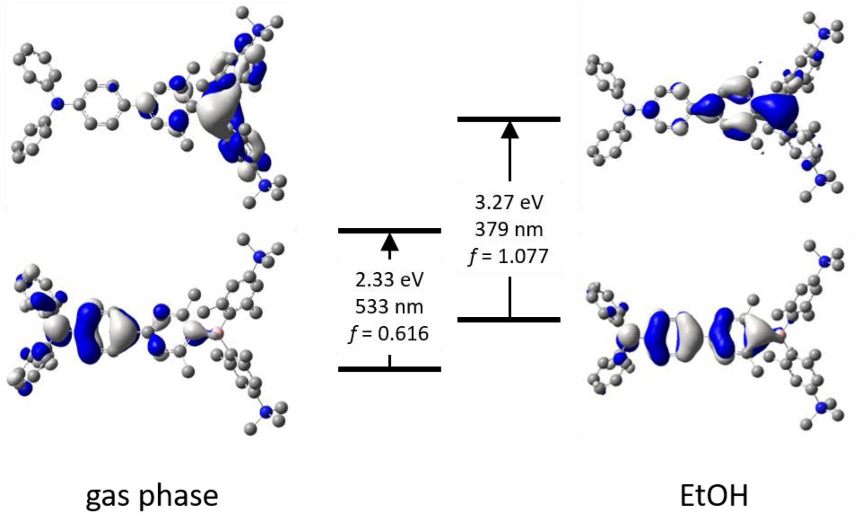

Figure S4. The natural transition orbitals (NTOs) of $S_1 \leftarrow S_0$ transition of compound **1M** are depicted based on TD-DFT calculations in the gas phase and in EtOH.

Co-localization with MitoTracker™ Deep Red

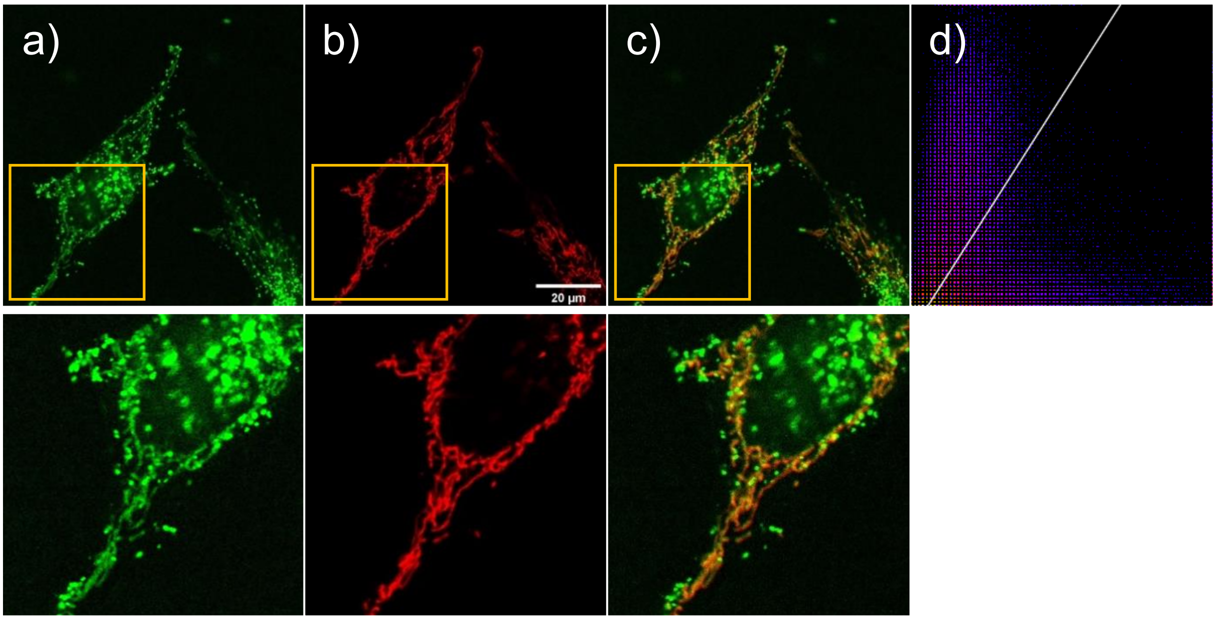

Figure S5. Co-staining experiment of HeLa cells with **1M** and MitoTracker™ Deep Red. The cells were loaded with **1M** (0.5 µM, 2 h) and MitoTracker™ Deep Red (0.05 µM, 20 min) at 37 °C. a) Imaging channel of **1M** (λ_{ex} = 405 nm; λ_{em} = 500 – 605 nm); b) imaging channel of MitoTracker™ Deep Red (λ_{ex} = 633 nm; λ_{em} = 645 – 786 nm); c) the merged fluorescence image of two channels; d) the correlation plot of the intensities (R_r = 0.42). Scale bar: 20 µm. The zoomed views of images (marked by square lines) are shown in the bottom.

NMR spectra

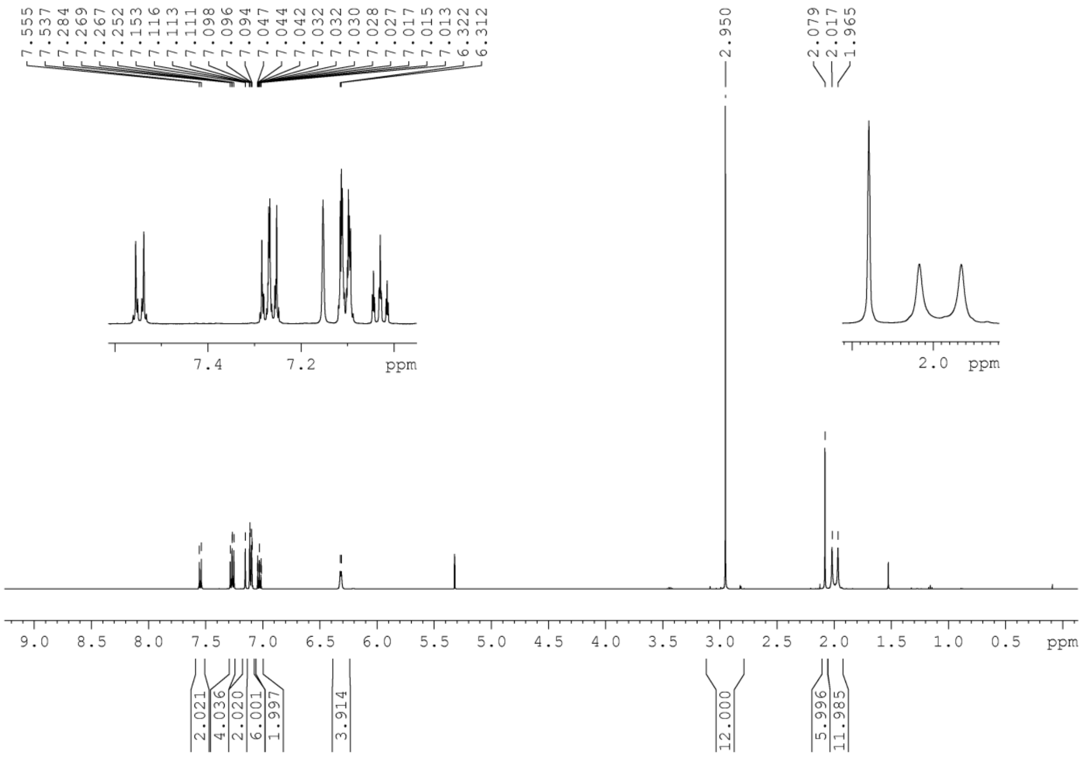

Figure S6. ^{1}H NMR spectrum of **1** in CD_2Cl_2 at 500 MHz.

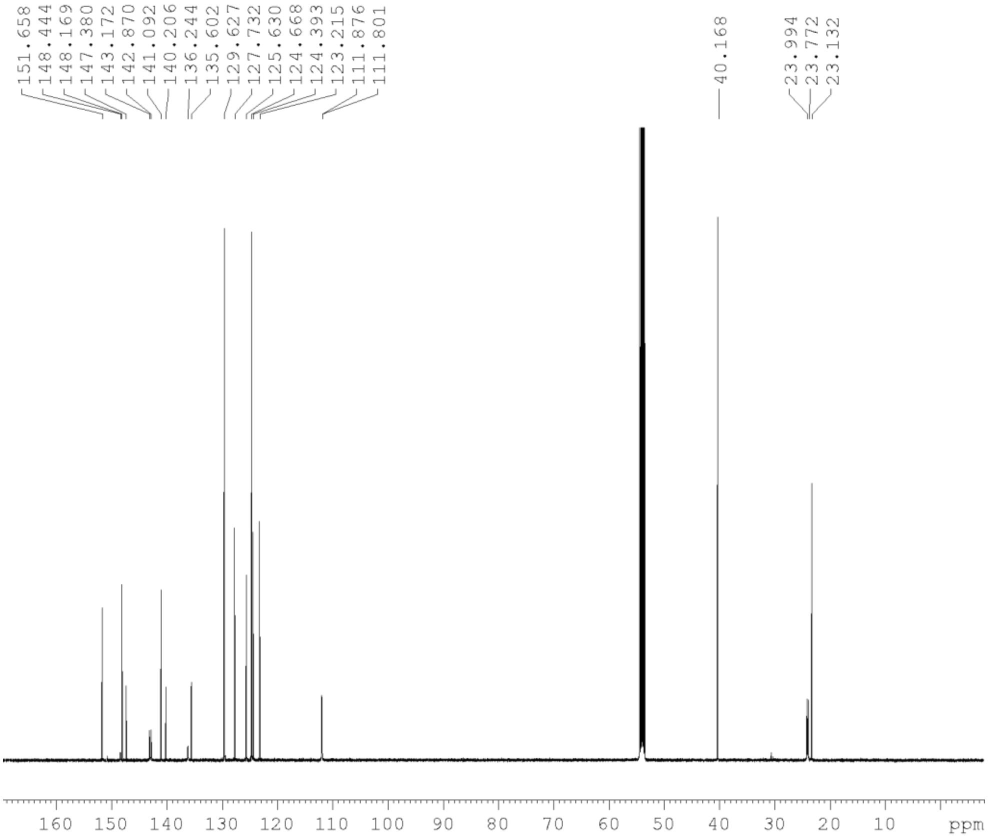

Figure S7. $^{13}C\{^{1}H\}$ NMR spectrum of **1** in CD_2Cl_2 at 125 MHz.

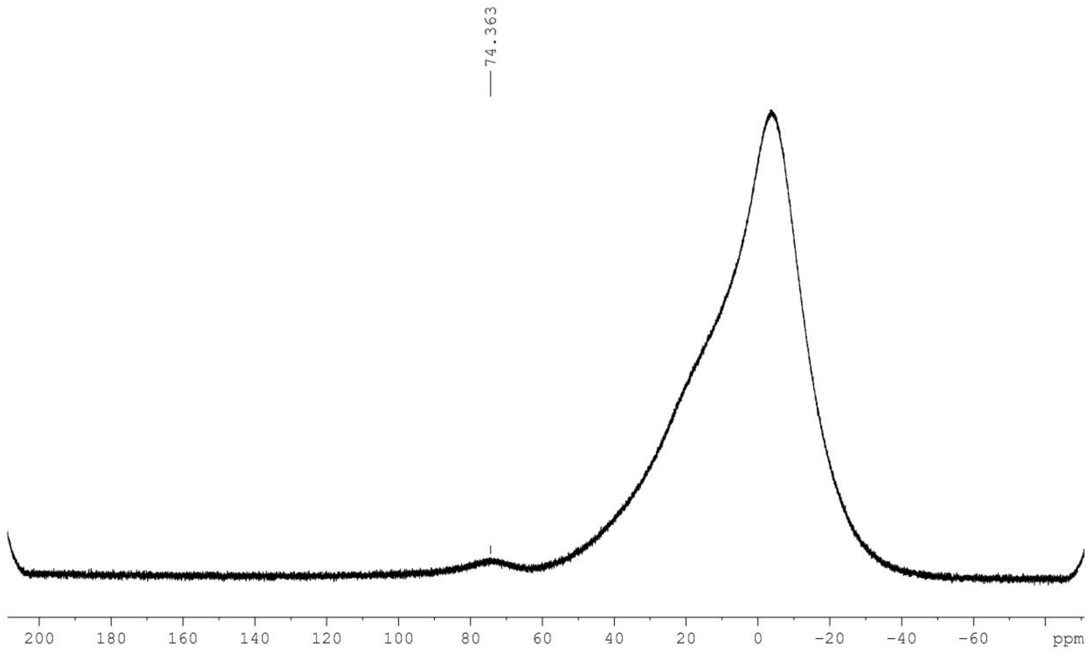

Figure S8. $^{11}B\{^{1}H\}$ NMR spectrum of **1** in CD_2Cl_2 at 160 MHz.

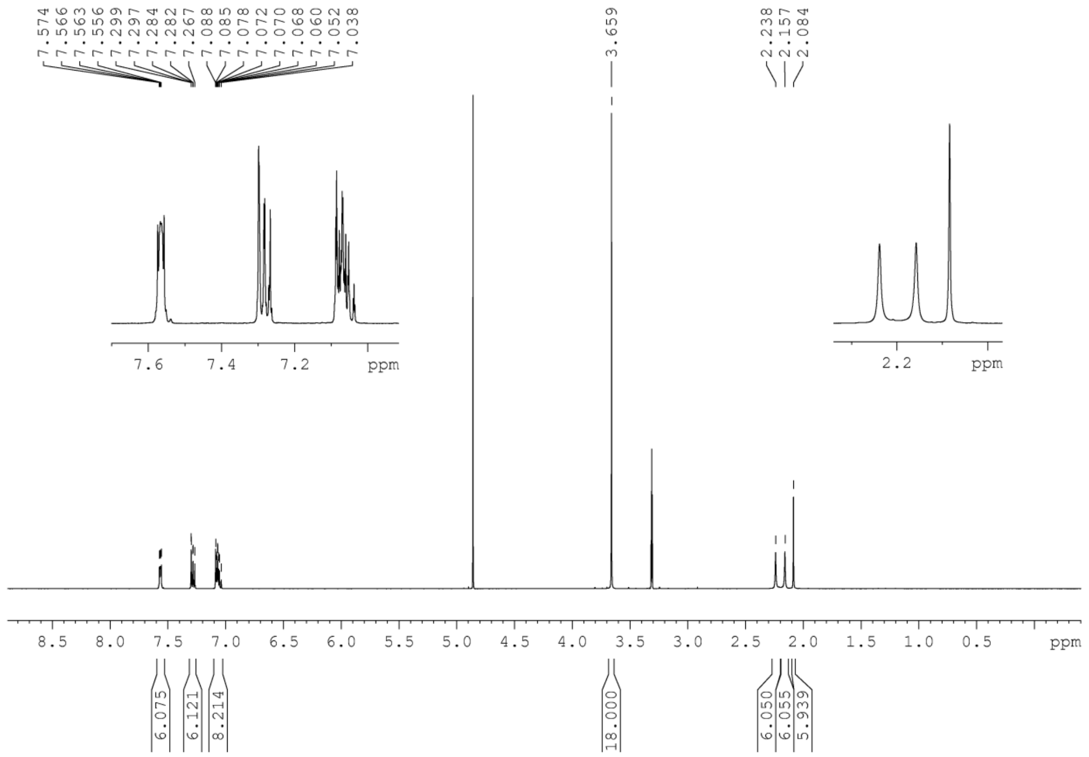

Figure S9. ^{1}H NMR spectrum of **1M** in CD_3OD at 500 MHz.

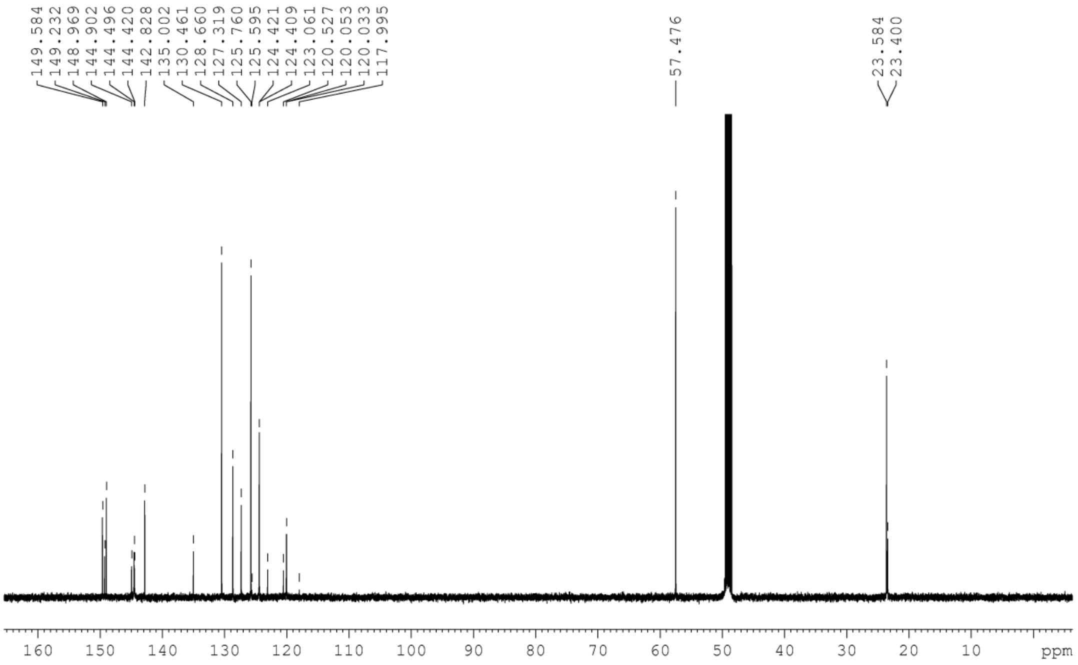

Figure S10. $^{13}C\{^{1}H\}$ NMR spectrum of **1M** in CD_3OD at 125 MHz.

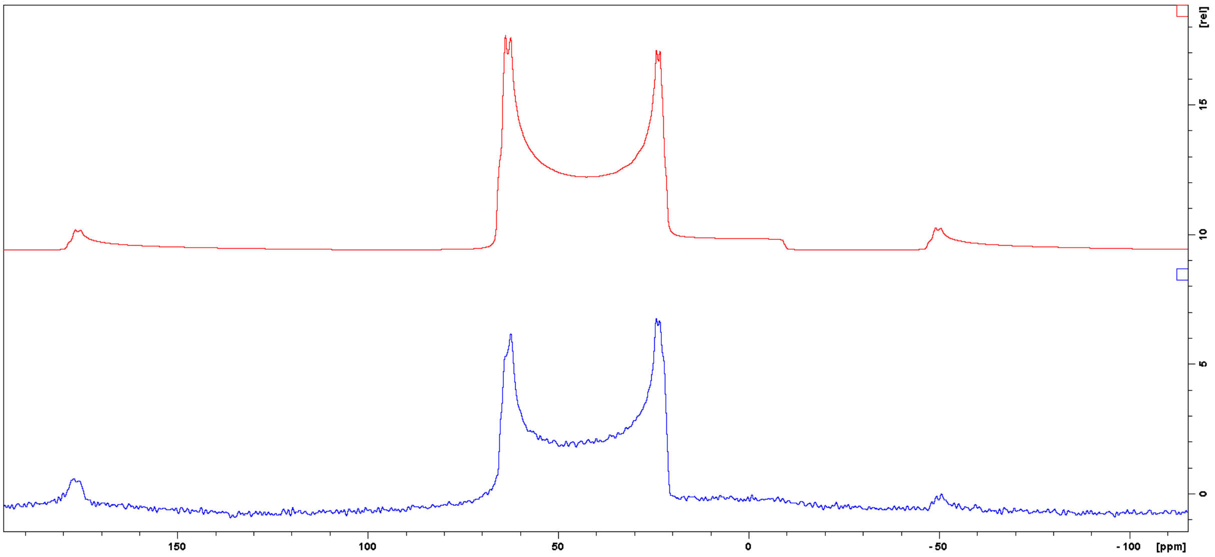

Figure S11. Solid-state $^{11}B\{^{1}H\}$ NMR spectrum of **1M** at 128 MHz. (Top: Simulation) isotropic chemical shift δ_{iso} = 76.0 and 77.6 ppm, quadrupolar coupling constant C_Q = 4.76 and 4.78 MHz, quadrupolar asymmetry parameter η_{Quad} = 0.0 and 0.0. Since no further side products are observable via NMR spectroscopy in solution, it can be assumed that the two signals correspond to two isomers, which exist in the solid state. An integration of the signals gives a ratio of 1:1.

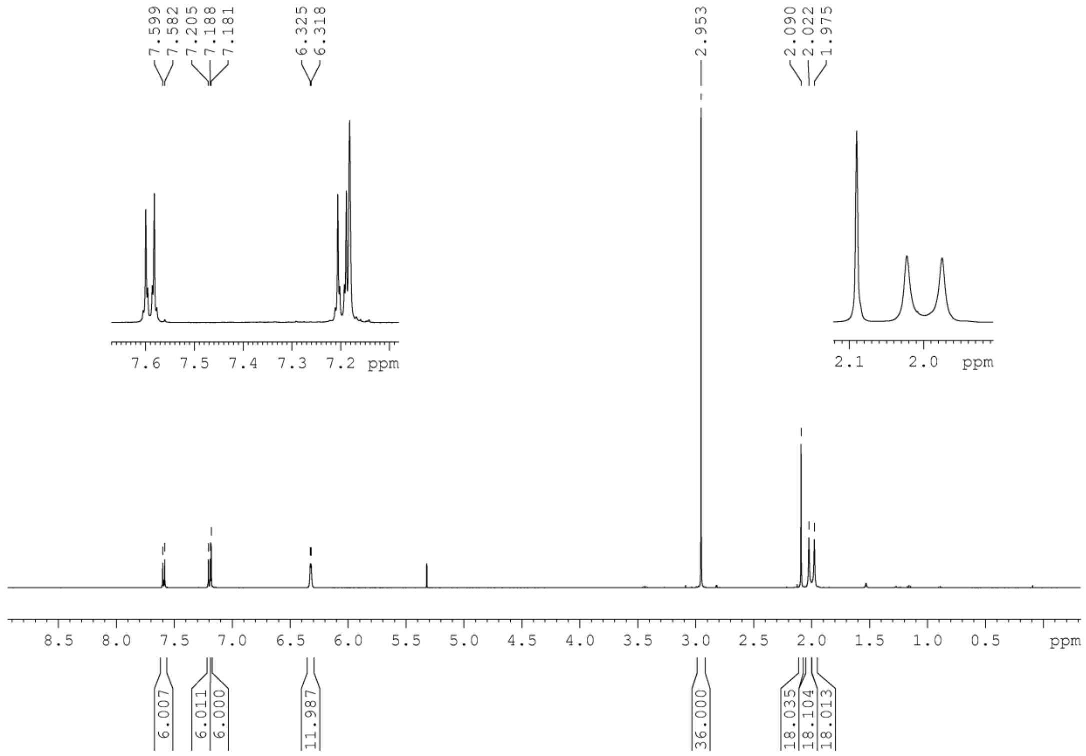

Figure S12. ^{1}H NMR spectrum of **2** in CD_2Cl_2 at 500 MHz.

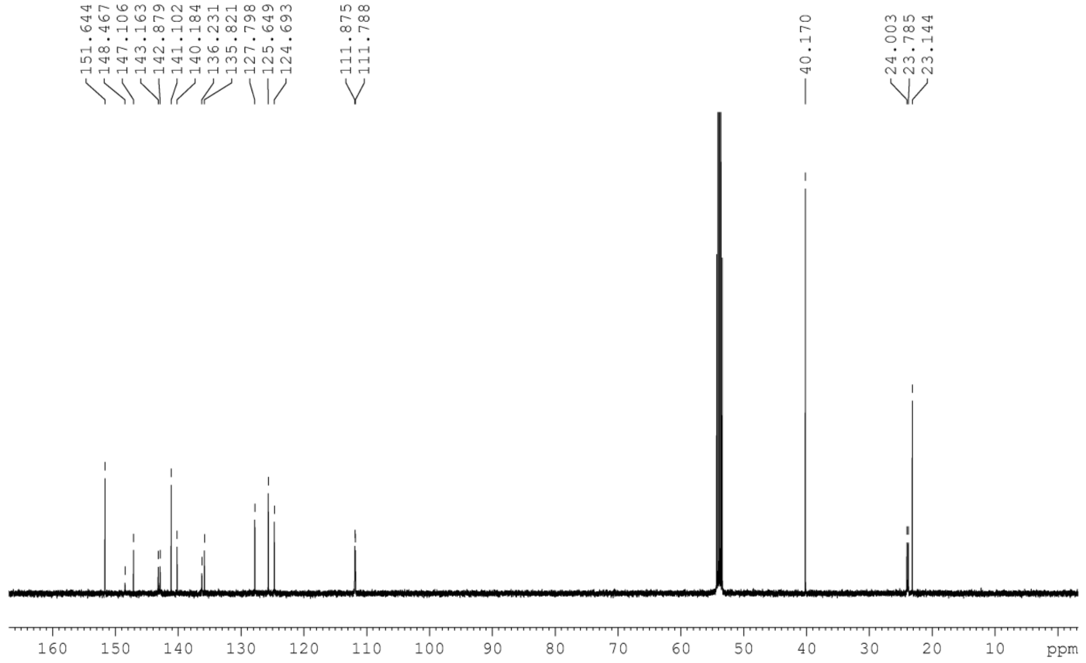

Figure S13. $^{13}C\{^{1}H\}$ NMR spectrum of **2** in CD_2Cl_2 at 125 MHz.

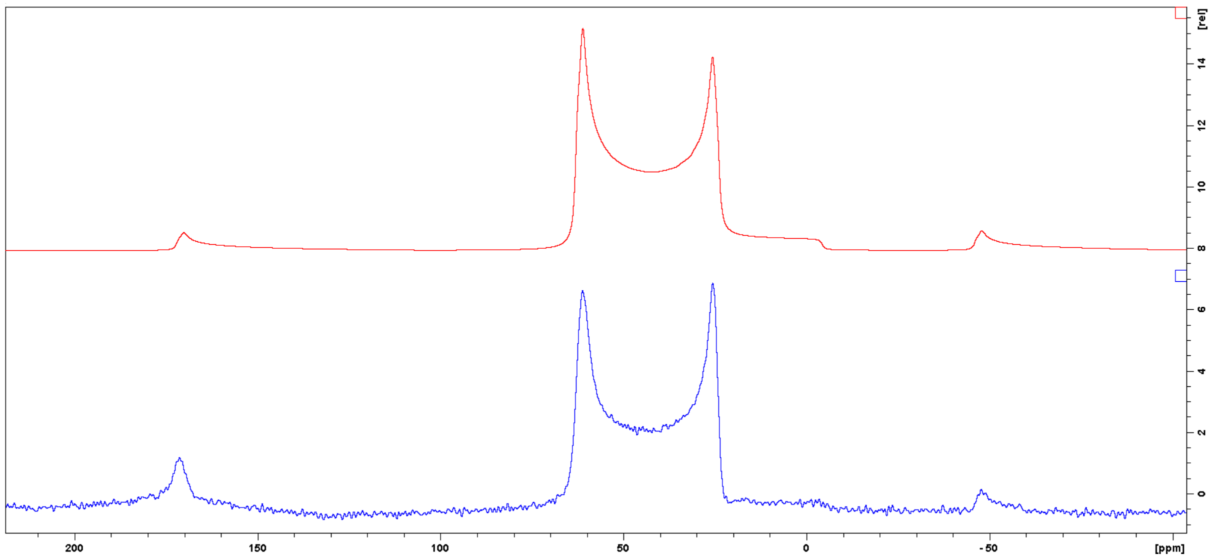

Figure S14. Solid-state $^{11}B\{^1H\}$ NMR spectrum of **2** at 128 MHz. (Top: Simulation) isotropic chemical shift δ_{iso} = 73.2 ppm, quadrupolar coupling constant C_Q = 4.53 MHz, quadrupolar asymmetry parameter η_{Quad} = 0.0.

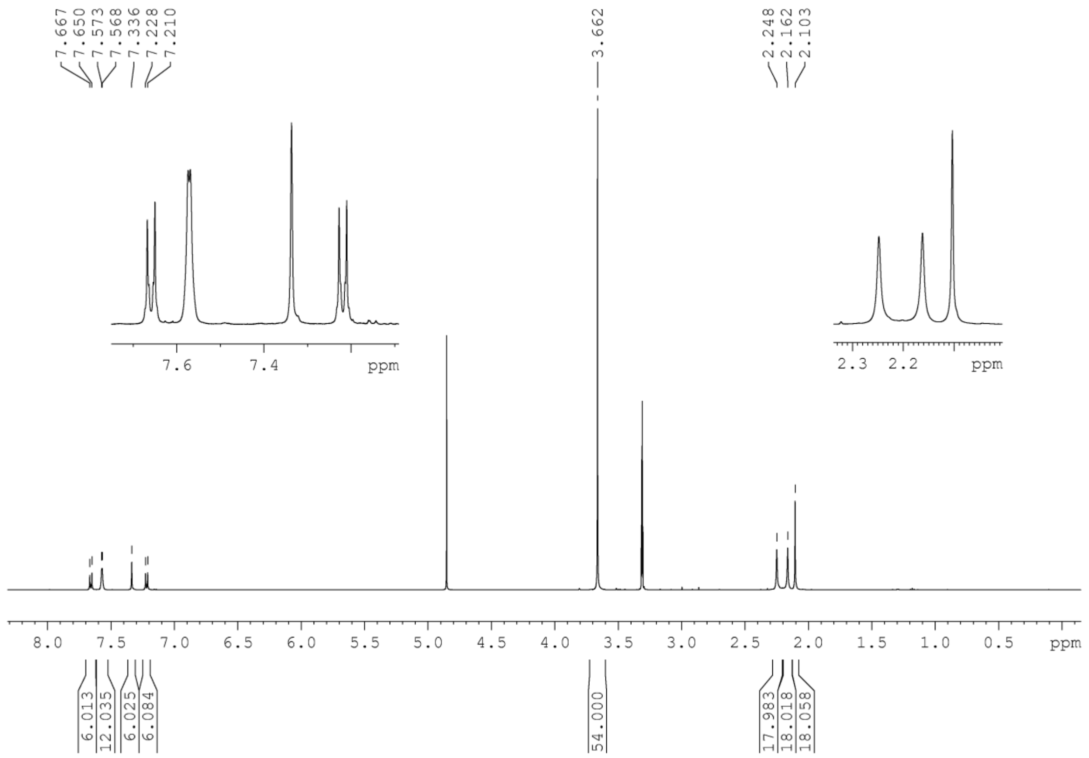

Figure S15. ^1H NMR spectrum of **2M** in CD_3OD at 500 MHz.

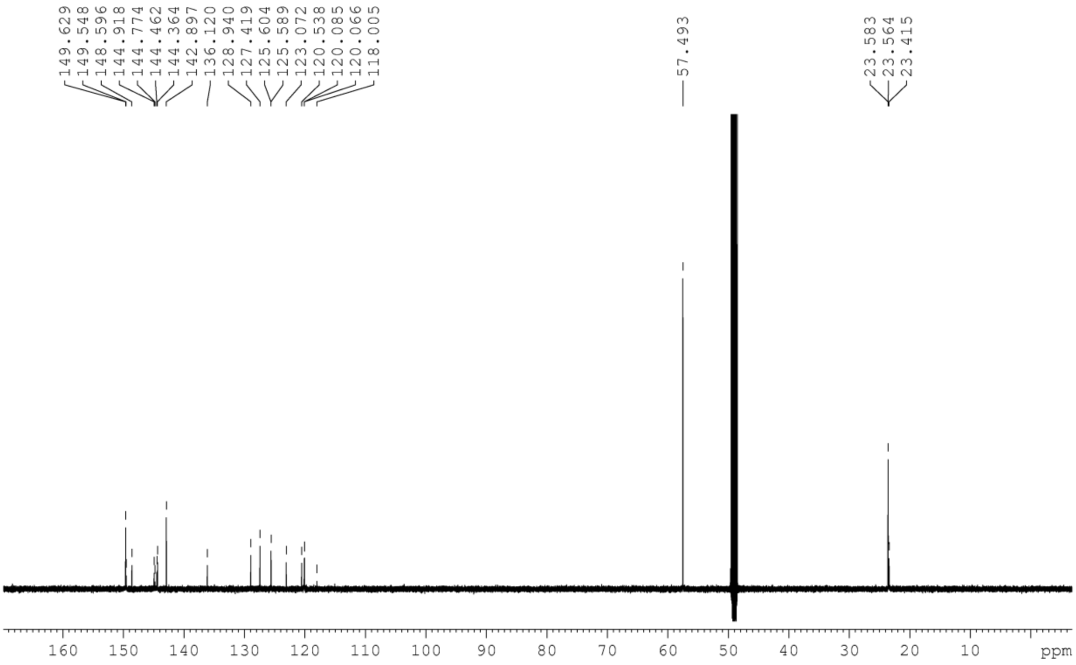

Figure S16. $^{13}C\{^1H\}$ NMR spectrum of **2M** in CD_3OD at 125 MHz.

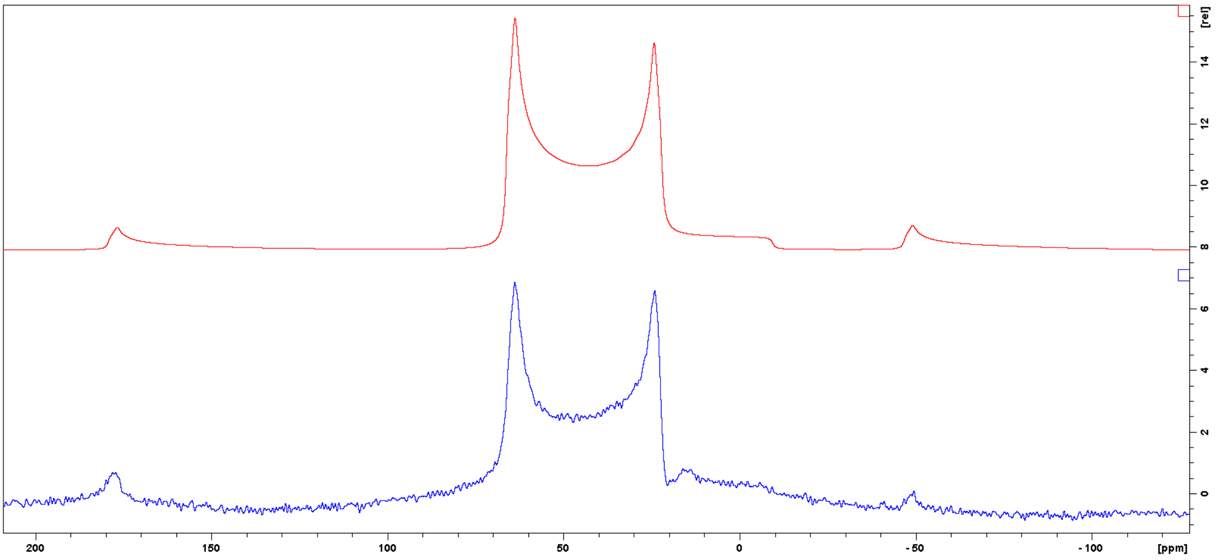

Figure S17. Solid-state $^{11}B\{^1H\}$ NMR spectrum of **2M** at 128 MHz. (Top: Simulation) isotropic chemical shift δ_{iso} = 77.6 ppm, quadrupolar coupling constant C_Q = 4.79 MHz, quadrupolar asymmetry parameter η_{Quad} = 0.1.

Cartesian coordinates for all DFT-optimized structures

Compound 1

DFT B3LYP/6-31G*, gas phase S_0

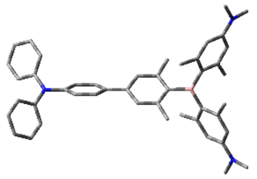

Point group: C_1

Total energy: -1237539.71 kcal mol^{-1}

Dipole moment: 2.66 D

Imaginary frequencies: 0

Symbol	X	Y	Z
N	-6.882105	0.000024	0.000068
C	-5.461794	0.000011	0.000029
C	-4.742346	0.206406	-1.188069
C	-4.742303	-0.206409	1.188099
C	-3.351855	0.213682	-1.180810
H	-5.280365	0.351133	-2.119607
C	-3.351814	-0.213713	1.180789
H	-5.280294	-0.351137	2.119654
C	-2.618801	-0.000019	-0.000025
H	-2.822651	0.347706	-2.120034
H	-2.822575	-0.347758	2.119990
C	-7.592965	-0.956888	0.774539
C	-8.733325	-0.580595	1.503115
C	-7.165948	-2.294041	0.824029
C	-9.431768	-1.524386	2.253654
H	-9.065887	0.452194	1.476245
C	-7.860625	-3.226396	1.592125
H	-6.289548	-2.594473	0.258726
C	-8.999517	-2.851213	2.308005
H	-10.311935	-1.214450	2.811167
H	-7.515213	-4.256720	1.618009
H	-9.542431	-3.582469	2.900063
C	-7.592993	0.956856	-0.774478
C	-8.733349	0.580501	-1.503016
C	-7.166002	2.294021	-0.824050
C	-9.431816	1.524237	-2.253615
H	-9.065902	-0.452289	-1.476082
C	-7.860698	3.226313	-1.592195
H	-6.289604	2.594497	-0.258765
C	-8.999591	2.851062	-2.308050
H	-10.311981	1.214242	-2.811099
H	-7.515316	4.256645	-1.618146
H	-9.542516	3.582277	-2.900149
C	-1.137192	-0.000032	-0.000050
C	-0.407879	-0.849195	0.842285
C	-0.407889	0.849127	-0.842397
H	-0.943430	-1.537721	1.491573
C	0.989345	-0.872743	0.845526
C	0.989336	0.872665	-0.845662
H	-0.943444	1.537662	-1.491671
C	1.726716	-0.000040	-0.000075
C	1.663934	-1.852220	1.788689
C	1.663915	1.852148	-1.788826
B	3.319410	-0.000014	-0.000035
H	2.144891	-2.673133	1.246932
H	2.444067	-1.378526	2.394895
H	0.931651	-2.286746	2.477459
H	2.144935	2.673025	-1.247072
H	2.443994	1.378435	-2.395089
H	0.931615	2.286723	-2.477548
C	4.099763	-1.356890	-0.201854
C	4.099669	1.356922	0.201828
C	5.184002	-1.720834	0.647516
C	3.750682	-2.289103	-1.220269
C	3.750553	2.289059	1.220293
C	5.183885	1.720950	-0.647527
C	5.853015	-2.934327	0.487849
C	5.653387	-0.843217	1.795432
C	4.456570	-3.480277	-1.385999
C	2.621062	-2.035026	-2.202742
C	4.456393	3.480259	1.386075
C	2.620970	2.034866	2.202777
C	5.852849	2.934466	-0.487805
C	5.653319	0.843377	-1.795456
C	5.526448	-3.838703	-0.542121
H	6.644997	-3.175265	1.187606
H	4.823858	-0.473476	2.406699
H	6.325274	-1.403107	2.454754
H	6.194071	0.039227	1.438248
H	4.160067	-4.140528	-2.192961
H	2.673276	-1.036208	-2.648661
H	2.653355	-2.761510	-3.021508
H	1.639845	-2.114466	-1.723940
C	5.526255	3.838770	0.542215
H	4.159852	4.140459	2.193065
H	1.639740	2.114247	1.723993
H	2.653223	2.761324	3.021566
H	2.673267	1.036037	2.648664
H	6.644826	3.175462	-1.187546
H	6.194042	-0.039050	-1.438285
H	4.823811	0.473609	-2.406734
H	6.325185	1.403308	-2.454766
N	6.228759	-5.022208	-0.721809
N	6.228567	5.022283	0.721977
C	5.720197	-6.022811	-1.644459
C	7.201295	-5.436276	0.274568
C	5.719876	6.022899	1.644551
C	7.200797	5.436571	-0.274615
H	5.660271	-5.624467	-2.664406
H	6.405652	-6.872585	-1.663146
H	4.720285	-6.392045	-1.365898
H	6.751991	-5.613731	1.265112
H	7.678400	-6.361641	-0.054849

H	7.988305	-4.682029	0.391610
H	5.659687	5.624495	2.664451
H	6.405427	6.872590	1.663457
H	4.720063	6.392262	1.365788
H	6.751217	5.614118	-1.265020
H	7.677914	6.361930	0.054803
H	7.987837	4.682406	-0.391956

Compound 2

DFT B3LYP/6-31G*, gas phase S_0

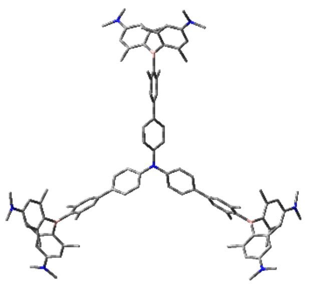

Point group: C_3

Total energy: -2771733.92 kcal mol^{-1}

Dipole moment: 0.00 D

Imaginary frequencies: 0

Symbol	X	Y	Z
C	2.603946	2.552778	0.782671
C	3.694074	2.129179	0.002286
C	3.514208	0.973509	-0.778031
C	2.308700	0.280253	-0.788704
C	1.230830	0.709341	0.002409
C	1.399849	1.857109	0.793496
N	0.000000	0.000000	0.002457
C	-1.229722	0.711260	0.002409
C	-0.001108	-1.420601	0.002409
C	-0.911644	-2.139519	-0.788704
C	-0.914021	-3.530147	-0.778031
C	-0.003114	-4.263751	0.002286
C	0.908798	-3.531472	0.782671
C	0.908379	-2.140859	0.793496
C	-2.308228	0.283750	0.793496
C	-3.512743	0.978694	0.782671
C	-3.690960	2.134573	0.002286
C	-2.600187	2.556639	-0.778031
C	-1.397056	1.859266	-0.788704
C	-0.003818	-5.746073	0.002125
C	-0.297421	-6.475265	-1.157377
C	-0.287783	-7.872468	-1.179763
C	-0.003158	-8.610169	0.001448
C	0.280406	-7.872891	1.183186
C	0.289430	-6.475675	1.161454
C	0.594632	-8.547543	2.506105
C	-0.602502	-8.546747	-2.502771
B	0.000000	-10.202587	0.000567
C	-0.860625	-10.986048	1.066722
C	0.864726	-10.979954	-1.066928
C	-0.311338	-12.069890	1.810312
C	-1.062971	-12.740669	2.775117
C	-2.409688	-12.416362	3.032756
C	-2.957214	-11.347264	2.296365
C	-2.211748	-10.639632	1.353904
C	0.319856	-12.063323	-1.814288
C	1.074646	-12.728577	-2.780486
C	2.420457	-12.398802	-3.035934
C	2.963677	-11.330242	-2.295568
C	2.214922	-10.628060	-1.351601
C	-1.113717	-12.535712	-1.641505
C	2.930670	-9.499453	-0.630551
C	1.123604	-12.537105	1.634874
C	-2.932083	-9.511005	0.637423
N	-3.163622	-13.120297	3.961676
N	3.177589	-13.097142	-3.966572
C	-4.465920	-12.612277	4.358056
C	4.478070	-12.582770	-4.360687
C	2.530038	-14.065701	-4.834075
C	-2.512447	-14.090413	4.824706
H	2.696070	3.452048	1.385028
H	4.338449	0.602520	-1.380539
H	2.199235	-0.606586	-1.404803
H	0.577624	2.206906	1.409639
H	-1.624936	-1.601300	-1.404803
H	-1.647427	-4.058468	-1.380539
H	1.641526	-4.060889	1.385028
H	1.622425	-1.603691	1.409639
H	-2.200049	-0.603216	1.409639
H	-4.337596	0.608841	1.385028
H	-2.691023	3.455947	-1.380539
H	-0.574299	2.207886	-1.404803
H	-0.507601	-5.939663	-2.080091
H	0.499252	-5.940366	2.084424
H	1.360112	-9.325235	2.407038
H	0.963387	-7.814665	3.231598
H	-0.288410	-9.031568	2.935761
H	-0.972558	-7.813843	-3.227585
H	0.280491	-9.029917	-2.933508
H	-1.367362	-9.325037	-2.403261
H	-0.580674	-13.532159	3.337175
H	-3.987992	-11.052586	2.456598
H	0.595519	-13.519998	-3.345335
H	3.993631	-11.031572	-2.453737
H	-1.829690	-11.707566	-1.633311
H	-1.396019	-13.208034	-2.458768
H	-1.253065	-13.076731	-0.699956
H	2.556960	-8.517821	-0.938854
H	2.807992	-9.555305	0.456223
H	4.004935	-9.529414	-0.840968
H	1.836788	-11.706600	1.627127
H	1.409154	-13.209906	2.450622
H	1.263510	-13.076078	0.692223
H	-2.561436	-8.529203	0.948874
H	-2.810349	-9.562481	-0.449667
H	-4.006023	-9.545393	0.848841

H	-4.412958	-11.612578	4.818050	H	6.098170	6.473303	-0.938854
H	-5.143958	-12.552076	3.498307	H	6.871141	7.209445	0.456223
H	-4.911915	-13.298343	5.081069	H	6.250247	8.233083	-0.840968
H	5.155494	-12.522930	-3.500445	N	12.944321	3.820371	3.961676
H	4.927026	-13.264677	-5.085812	N	9.753663	9.300444	-3.966572
H	4.421477	-11.581706	-4.817289	C	13.155512	2.438539	4.358056
H	1.766557	-13.613729	-5.487872	C	13.458879	4.869364	4.824706
H	3.284058	-14.539409	-5.466190	C	8.657963	10.169507	-4.360687
H	2.046655	-14.855862	-4.247623	C	10.916235	9.223927	-4.834075
H	-2.027100	-14.876669	4.234633	H	12.263267	1.984556	4.818050
H	-1.749850	-13.638750	5.479732	H	13.442396	1.821240	3.498307
H	-3.264518	-14.568967	5.455482	H	13.972661	2.395329	5.081069
C	4.978154	2.869730	0.002125	H	13.897123	5.682815	4.234633
C	5.756454	2.980058	-1.157377	H	12.686429	5.303961	5.479732
C	5.463384	3.488491	1.161454	H	14.249354	4.457328	5.455482
C	6.961649	3.687006	-1.179763	H	8.267429	10.726253	-3.500445
H	5.397700	2.530236	-2.080091	H	9.024034	10.899268	-5.085812
C	6.677920	4.179284	1.183186	H	7.819313	9.619965	-4.817289
H	4.894882	3.402548	2.084424	H	10.906557	8.336748	-5.487872
C	7.458204	4.302349	0.001448	H	10.949469	10.113782	-5.466190
C	7.702951	3.751592	-2.502771	H	11.842226	9.200386	-4.247623
C	7.105074	4.788738	2.506105	C	-4.974336	2.876343	0.002125
B	8.835699	5.101293	0.000567	C	-5.459033	3.495206	-1.157377
H	7.253266	3.064661	-3.227585	C	-5.752814	2.987184	1.161454
H	7.679892	4.757871	-2.933508	C	-6.673865	4.185461	-1.179763
H	8.759400	3.478349	-2.403261	H	-4.890098	3.409427	-2.080091
H	7.395834	5.840509	2.407038	C	-6.958326	3.693606	1.183186
H	6.286005	4.741650	3.231598	H	-5.394134	2.537818	2.084424
H	7.965772	4.266014	2.935761	C	-7.455046	4.307819	0.001448
C	9.944509	4.747701	1.066722	C	-7.100449	4.795155	-2.502771
C	9.076556	6.238852	-1.066928	C	-7.699705	3.758805	2.506105
C	10.608501	5.765319	1.810312	B	-8.835699	5.101293	0.000567
C	10.320066	3.404387	1.353904	H	-6.280707	4.749182	-3.227585
C	10.287216	6.308665	-1.814288	H	-7.960383	4.272046	-2.933508
C	8.096709	7.232209	-1.351601	H	-7.392038	5.846688	-2.403261
C	11.565228	5.449774	2.775117	H	-8.755946	3.484725	2.407038
C	10.295650	7.241622	1.634874	H	-7.249392	3.073015	3.231598
C	11.305626	3.112610	2.296365	H	-7.677363	4.765554	2.935761
C	9.702813	2.216244	0.637423	C	-9.083885	6.238347	1.066722
C	10.485948	7.294960	-2.780486	C	-9.941282	4.741102	-1.066928
C	11.413103	5.303348	-1.641505	C	-10.297163	6.304572	1.810312
C	8.330439	8.231741	-2.295568	C	-8.108318	7.235246	1.353904
C	6.761433	7.287761	-0.630551	C	-10.607072	5.754658	-1.814288
C	11.957729	4.121330	3.032756	C	-10.311631	3.395851	-1.351601
H	12.009531	6.263201	3.337175	C	-10.502257	7.290894	2.775117
H	9.219819	7.444005	1.627127	C	-11.419253	5.295483	1.634874
H	10.735537	7.825316	2.450622	C	-8.348412	8.234655	2.296365
H	10.692461	7.632270	0.692223	C	-6.770730	7.294761	0.637423
H	11.565816	2.072590	2.456598	C	-11.560595	5.433618	-2.780486
H	8.667224	2.046332	0.948874	C	-10.299386	7.232363	-1.641505
H	9.686526	2.347407	-0.449667	C	-11.294116	3.098501	-2.295568
H	10.269564	1.303379	0.848841	C	-9.692103	2.211692	-0.630551
C	9.527449	8.295579	-3.035934	C	-9.548041	8.295032	3.032756
H	11.410902	7.275734	-3.345335	H	-11.428856	7.268958	3.337175
H	11.053895	4.269225	-1.633311	H	-11.056607	4.262595	1.627127
H	12.136502	5.395029	-2.458768	H	-12.144691	5.384590	2.450622
H	11.951314	5.453179	-0.699956	H	-11.955970	5.443807	0.692223
H	7.556806	8.974372	-2.453737	H	-7.577824	8.979996	2.456598

H	-6.105788	6.482870	0.948874
H	-6.876177	7.215074	-0.449667
H	-6.263541	8.242014	0.848841
C	-11.947906	4.103224	-3.035934
H	-12.006421	6.244264	-3.345335
H	-9.224205	7.438341	-1.633311
H	-10.740483	7.813005	-2.458768
H	-10.698249	7.623552	-0.699956
H	-11.550437	2.057200	-2.453737
H	-8.655130	2.044519	-0.938854
H	-9.679132	2.345860	0.456223
H	-10.255182	1.296331	-0.840968
N	-9.780699	9.299926	3.961676
N	-12.931252	3.796698	-3.966572
C	-8.689593	10.173738	4.358056
C	-10.946433	9.221049	4.824706
C	-13.136033	2.413263	-4.360687
C	-13.446273	4.841773	-4.834075
H	-7.850309	9.628023	4.818050
H	-8.298438	10.730837	3.498307
H	-9.060746	10.903015	5.081069
H	-11.870024	9.193854	4.234633
H	-10.936579	8.334789	5.479732
H	-10.984836	10.111639	5.455482
H	-13.422923	1.796677	-3.500445
H	-13.951060	2.365409	-5.085812
H	-12.240790	1.961742	-4.817289
H	-12.673114	5.276982	-5.487872
H	-14.233527	4.425627	-5.466190
H	-13.888881	5.655476	-4.247623

Compound 1M

DFT B3LYP/6-31G*, gas phase S_0

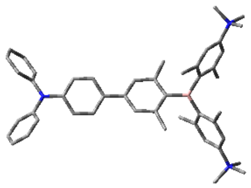

Point group: C_2

Total energy: -1287314.85 kcal mol^{-1}

Imaginary frequencies: 0

Symbol	X	Y	Z
C	2.309186	2.669883	8.380457
C	2.989741	1.995775	9.396774
C	2.681871	0.660486	9.666285
C	1.707497	-0.002687	8.921772
C	1.024189	0.674454	7.903505
C	1.323758	2.018237	7.639942
N	0.000000	0.000000	7.161104
C	0.000000	0.000000	5.769790
C	-1.024189	-0.674454	7.903505
C	-1.323758	-2.018237	7.639942
C	-2.309186	-2.669883	8.380457
C	-2.989741	-1.995775	9.396774
C	-2.681871	-0.660486	9.666285
C	-1.707497	0.002687	8.921772
C	-1.192969	-0.201384	5.036453
C	-1.183370	-0.205961	3.653245
C	0.000000	0.000000	2.910922
C	1.183370	0.205961	3.653245
C	1.192969	0.201384	5.036453
C	0.000000	0.000000	1.446414
C	0.983333	0.681671	0.702019
C	1.016168	0.677748	-0.687153
C	0.000000	0.000000	-1.444263
C	-1.016168	-0.677748	-0.687153
C	-0.983333	-0.681671	0.702019
C	-2.129743	-1.488389	-1.327349
C	2.129743	1.488389	-1.327349
B	0.000000	0.000000	-2.993712
C	-1.373852	0.119983	-3.818787
C	1.373852	-0.119983	-3.818787
C	-1.762064	-0.822296	-4.802693
C	-2.986126	-0.697740	-5.479780
C	-3.826882	0.373148	-5.206469
C	-3.456229	1.329755	-4.265441
C	-2.250804	1.211526	-3.563730
C	1.762064	0.822296	-4.802693
C	2.986126	0.697740	-5.479780
C	3.826882	-0.373148	-5.206469
C	3.456229	-1.329755	-4.265441
C	2.250804	-1.211526	-3.563730
C	0.910689	2.027524	-5.143562
C	1.953668	-2.281387	-2.532049
C	-0.910689	-2.027524	-5.143562
C	-1.953668	2.281387	-2.532049
N	-5.161908	0.532157	-5.905961
N	5.161908	-0.532157	-5.905961
C	-5.439823	-0.571814	-6.897011
C	-6.279023	0.514770	-4.879590
C	5.439823	0.571814	-6.897011
C	6.279023	-0.514770	-4.879590
C	-5.192094	1.844673	-6.664088
C	5.192094	-1.844673	-6.664088
H	2.532288	3.712822	8.173468
H	3.749899	2.508831	9.978336
H	3.205708	0.128649	10.455350
H	1.468646	-1.041500	9.127173
H	0.778863	2.546350	6.863170
H	-0.778863	-2.546350	6.863170
H	-2.532288	-3.712822	8.173468
H	-3.749899	-2.508831	9.978336
H	-3.205708	-0.128649	10.455350
H	-1.468646	1.041500	9.127173
H	-2.129263	-0.332169	5.566369
H	-2.131195	-0.322688	3.136094
H	2.131195	0.322688	3.136094
H	2.129263	0.332169	5.566369
H	1.726916	1.267910	1.232557

H	-1.726916	-1.267910	1.232557
H	-1.773078	-2.137482	-2.133424
H	-2.604185	-2.131585	-0.580778
H	-2.914210	-0.850583	-1.750024
H	2.604185	2.131585	-0.580778
H	2.914210	0.850583	-1.750024
H	1.773078	2.137482	-2.133424
H	-3.243352	-1.458726	-6.205088
H	-4.094127	2.178950	-4.041884
H	3.243352	1.458726	-6.205088
H	4.094127	-2.178950	-4.041884
H	0.714356	2.645228	-4.260117
H	1.404102	2.666785	-5.881353
H	-0.059715	1.731288	-5.552781
H	2.096061	-1.900511	-1.515665
H	0.922527	-2.640204	-2.587441
H	2.613600	-3.144308	-2.663428
H	-0.714356	-2.645228	-4.260117
H	-1.404102	-2.666785	-5.881353
H	0.059715	-1.731288	-5.552781
H	-2.096061	1.900511	-1.515665
H	-0.922527	2.640204	-2.587441
H	-2.613600	3.144308	-2.663428
H	-6.413246	-0.378537	-7.348377
H	-4.668895	-0.567981	-7.666741
H	-5.458586	-1.528176	-6.375685
H	-7.232441	0.617853	-5.400648
H	-6.142797	1.340425	-4.184048
H	-6.235941	-0.433390	-4.343315
H	6.413246	0.378537	-7.348377
H	4.668895	0.567981	-7.666741
H	5.458586	1.528176	-6.375685
H	7.232441	-0.617853	-5.400648
H	6.142797	-1.340425	-4.184048
H	6.235941	0.433390	-4.343315
H	-4.381727	1.837474	-7.393315
H	-6.157308	1.935839	-7.165234
H	-5.056082	2.668305	-5.966118
H	4.381727	-1.837474	-7.393315
H	6.157308	-1.935839	-7.165234
H	5.056082	-2.668305	-5.966118

Compound 2M

DFT B3LYP/6-31G*, gas phase S_0

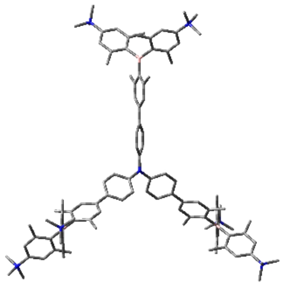

Point group: C_3

Total energy: -2920867.63 kcal mol^{-1}

Imaginary frequencies: 0

Symbol	X	Y	Z
C	-2.600782	-2.567871	0.763348
C	-3.700035	-2.136216	-0.002780
C	-3.524694	-0.968854	-0.769706
C	-2.320048	-0.273682	-0.779223
C	-1.232619	-0.711778	-0.004029
C	-1.396365	-1.872304	0.771670
N	0.000000	0.000000	-0.004513
C	1.232727	-0.711590	-0.004029
C	-0.000108	1.423368	-0.004029
C	0.923009	2.146061	-0.779223
C	0.923294	3.536901	-0.769706
C	0.000001	4.272432	-0.002780
C	-0.923451	3.536279	0.763348
C	-0.923281	2.145440	0.771670
C	2.319645	-0.273135	0.771670
C	3.524233	-0.968408	0.763348
C	3.700035	-2.136217	-0.002780
C	2.601399	-2.568047	-0.769706
C	1.397039	-1.872380	-0.779223
C	0.000157	5.755977	-0.001802
C	0.397681	6.492416	-1.128353
C	0.391444	7.888076	-1.155142
C	0.000000	8.635308	0.000284
C	-0.390604	7.886367	1.155022
C	-0.396828	6.490750	1.126031
C	-0.845998	8.535036	2.449970
C	0.847587	8.538700	-2.448848
B	-0.002884	10.204008	0.000352
C	0.535873	11.044427	1.258117
C	-0.546663	11.041099	-1.257305
C	-0.243791	12.051709	1.879398
C	0.267662	12.798858	2.953359
C	1.559806	12.575229	3.409263
C	2.346768	11.592268	2.813669
C	1.847917	10.817732	1.759144
C	0.228671	12.056579	-1.879802
C	-0.292487	12.796762	-2.947035
C	-1.587796	12.567121	-3.403690

C	-2.366995	11.581917	-2.811332
C	-1.854410	10.808612	-1.755471
C	1.646376	12.375471	-1.451509
C	-2.766132	9.732910	-1.197696
C	-1.662207	12.366787	1.448852
C	2.765354	9.749441	1.197861
H	-2.679229	-3.473971	1.356621
H	-4.349007	-0.583980	-1.362610
H	-2.220098	0.618913	-1.387977
H	-0.572948	-2.231873	1.379935
H	1.646043	1.613205	-1.387977
H	1.668762	4.058341	-1.362610
H	-1.668933	4.057266	1.356621
H	-1.646384	1.612124	1.379935
H	2.219332	0.619749	1.379935
H	4.348162	-0.583295	1.356621
H	2.680245	-3.474361	-1.362610
H	0.574055	-2.232117	-1.387977
H	0.696114	5.961523	-2.027793
H	-0.694629	5.958445	2.024831
H	-1.583078	9.328478	2.290500
H	-1.310020	7.791786	3.104396
H	-0.010609	8.978313	3.002712
H	1.321948	7.798700	-3.099574
H	0.010778	8.972913	-3.006691
H	1.576316	9.339214	-2.286897
H	-0.374755	13.546462	3.400007
H	3.358392	11.397694	3.154633
H	0.339518	13.553007	-3.400944
H	-3.376373	11.375943	-3.142940
H	2.265815	11.475511	-1.381646
H	2.132492	13.046019	-2.165665
H	1.669113	12.862362	-0.471465
H	-2.432000	8.735692	-1.502705
H	-2.797102	9.732514	-0.104495
H	-3.791506	9.865928	-1.554735
H	-2.271752	11.463525	1.346956
H	-2.161079	13.011065	2.178253
H	-1.682312	12.882979	0.483569
H	2.421365	8.747755	1.475101
H	2.816716	9.772633	0.105363
H	3.784400	9.870932	1.576443
C	-4.984900	-2.877853	-0.001802
C	-5.821437	-2.901806	-1.128353
C	-5.422740	-3.589038	1.126031
C	-7.026996	-3.605038	-1.155142
H	-5.510888	-2.377909	-2.027793
C	-6.634492	-4.281456	1.155022
H	-4.812850	-3.580789	2.024831
C	-7.478396	-4.317654	0.000284
C	-7.818524	-3.535318	-2.448848
C	-6.968559	-5.000174	2.449970
B	-8.835488	-5.104502	0.000352
H	-7.414846	-2.754509	-3.099574
H	-7.776160	-4.477122	-3.006691
H	-8.876155	-3.304477	-2.286897
H	-7.287160	-6.035224	2.290500
H	-6.092875	-5.030403	3.104396
H	-7.770142	-4.498345	3.002712
C	-9.832691	-5.058134	1.258117
C	-9.288541	-5.993974	-1.257305
C	-10.315191	-6.236984	1.879398
C	-10.292389	-3.808523	1.759144
C	-10.555639	-5.830254	-1.879802
C	-8.433328	-7.010272	-1.755471
C	-11.217967	-6.167627	2.953359
C	-9.878848	-7.622907	1.448852
C	-11.212582	-3.763774	2.813669
C	-9.825941	-2.479853	1.197861
C	-10.936078	-6.651682	-2.947035
C	-11.540661	-4.761932	-1.451509
C	-8.846737	-7.840836	-2.811332
C	-7.045881	-7.261996	-1.197696
C	-11.670371	-4.936783	3.409263
H	-11.544203	-7.097778	3.400007
H	-8.791828	-7.699157	1.346956
H	-10.187373	-8.377082	2.178253
H	-10.315831	-7.898414	0.483569
H	-11.549888	-2.790394	3.154633
H	-8.786460	-2.276914	1.475101
H	-9.871706	-2.446969	0.105363
H	-10.440677	-1.658079	1.576443
C	-10.089548	-7.658632	-3.403690
H	-11.907007	-6.482472	-3.400944
H	-11.070991	-3.775503	-1.381646
H	-12.364430	-4.676217	-2.165665
H	-11.973688	-4.985687	-0.471465
H	-8.163669	-8.611996	-3.142940
H	-6.349332	-6.474020	-1.502705
H	-7.030053	-7.288618	-0.104495
H	-6.648391	-8.216504	-1.554735
C	4.984744	-2.878124	-0.001802
C	5.423756	-3.590610	-1.128353
C	5.819568	-2.901712	1.126031
C	6.635552	-4.283039	-1.155142
H	4.814774	-3.583614	-2.027793
C	7.025096	-3.604911	1.155022
H	5.507479	-2.377656	2.024831
C	7.478396	-4.317654	0.000284
C	6.970937	-5.003382	-2.448848
C	7.814557	-3.534862	2.449970
B	8.838372	-5.099506	0.000352
H	6.092898	-5.044191	-3.099574
H	7.765381	-4.495791	-3.006691
H	7.299838	-6.034737	-2.286897
H	8.870238	-3.293254	2.290500
H	7.402895	-2.761383	3.104396
H	7.780752	-4.479969	3.002712
C	9.296818	-5.986293	1.258117
C	9.835204	-5.047125	-1.257305
C	10.558982	-5.814725	1.879398
C	8.444472	-7.009209	1.759144
C	10.326968	-6.226325	-1.879802
C	10.287738	-3.798340	-1.755471
C	10.950305	-6.631231	2.953359
C	11.541055	-4.743880	1.448852

C	8.865815	-7.828494	2.813669
C	7.060587	-7.269588	1.197861
C	11.228565	-6.145080	-2.947035
C	9.894285	-7.613539	-1.451509
C	11.213732	-3.741081	-2.811332
C	9.812013	-2.470914	-1.197696
C	10.110565	-7.638447	3.409263
H	11.918958	-6.448684	3.400007
H	11.063580	-3.764368	1.346956
H	12.348452	-4.633983	2.178253
H	11.998143	-4.984565	0.483569
H	8.191497	-8.607299	3.154633
H	6.365096	-6.470841	1.475101
H	7.054990	-7.325664	0.105363
H	6.656278	-8.212852	1.576443
C	11.677344	-4.908489	-3.403690
H	11.567489	-7.070534	-3.400944
H	8.805177	-7.700009	-1.381646
H	10.231938	-8.369802	-2.165665
H	10.304576	-7.876675	-0.471465
H	11.540042	-2.763946	-3.142940
H	8.781331	-2.261673	-1.502705
H	9.827155	-2.443896	-0.104495
H	10.439897	-1.649424	-1.554735
N	12.668081	-4.878700	-4.549361
C	13.074888	-3.476615	-4.938884
C	13.930283	-5.625693	-4.157385
C	12.055037	-5.532417	-5.774703
H	12.194911	-2.922087	-5.262986
H	13.548814	-2.988206	-4.088088
H	13.785711	-3.552247	-5.761975
H	14.355873	-5.142464	-3.277659
H	13.682938	-6.661280	-3.932401
H	14.631786	-5.584184	-4.992187
H	11.811957	-6.568814	-5.548704
H	11.149722	-4.985331	-6.038550
H	12.779242	-5.489602	-6.589842
N	10.512631	-8.546796	4.553085
C	11.884254	-8.229383	5.103648
C	9.521263	-8.405213	5.693283
C	10.535656	-9.988888	4.079540
H	12.627353	-8.353400	4.316691
H	11.892876	-7.209826	5.487472
H	12.087947	-8.929042	5.914472
H	9.518222	-7.364427	6.017831
H	8.529797	-8.691563	5.347865
H	9.834793	-9.058745	6.508972
H	9.543116	-10.272715	3.735122
H	11.252597	-10.070392	3.262313
H	10.836002	-10.623892	4.914583
N	-2.108963	13.410229	-4.549361
C	-3.526607	13.061492	-4.938884

C	-2.093148	14.876826	-4.157385
C	-1.236305	13.206176	-5.774703
H	-3.566854	12.022146	-5.262986
H	-4.186545	13.227720	-4.088088
H	-3.816519	13.714900	-5.761975
H	-2.724432	15.003783	-3.277659
H	-1.072631	15.180412	-3.932401
H	-2.479848	15.463590	-4.992187
H	-0.217219	13.513862	-5.548704
H	-1.257438	12.148608	-6.038550
H	-1.635487	13.811949	-6.589842
N	2.145427	13.377604	4.553085
C	1.184728	14.406757	5.103648
C	2.518497	12.448262	5.693283
C	3.382803	14.118590	4.079540
H	0.920581	15.112309	4.316691
H	0.297454	13.904446	5.487472
H	1.688804	14.932990	5.914472
H	1.618670	11.925236	6.017831
H	3.262216	11.732802	5.347865
H	2.927707	13.046553	6.508972
H	4.124874	13.400939	3.735122
H	3.094917	14.780231	3.262313
H	3.782559	14.696199	4.914583
N	-10.559118	-8.531530	-4.549361
C	-11.837135	-9.251132	-4.157385
C	-10.818732	-7.673760	-5.774703
C	-9.548281	-9.584878	-4.938884
H	-11.631441	-9.861318	-3.277659
H	-12.610307	-8.519132	-3.932401
H	-12.151938	-9.879407	-4.992187
H	-11.594738	-6.945048	-5.548704
H	-9.892284	-7.163277	-6.038550
H	-11.143756	-8.322348	-6.589842
H	-8.628057	-9.100059	-5.262986
H	-9.362269	-10.239514	-4.088088
H	-9.969192	-10.162653	-5.761975
N	-12.658058	-4.830808	4.553085
C	-13.068982	-6.177375	5.103648
C	-12.039760	-4.043049	5.693283
C	-13.918459	-4.129701	4.079540
H	-13.547933	-6.758908	4.316691
H	-12.190330	-6.694620	5.487472
H	-13.776750	-6.003948	5.914472
H	-11.136892	-4.560809	6.017831
H	-11.792013	-3.041239	5.347865
H	-12.762500	-3.987808	6.508972
H	-13.667990	-3.128224	3.735122
H	-14.347513	-4.709839	3.262313
H	-14.618561	-4.072307	4.914583

References

[1] S. S. Zalesskiy, V. P. Ananikov, *Organometallics* **2012**, *31*, 2302-2309.

[2] S. Griesbeck, Z. Zhang, M. Gutmann, T. Lühmann, R. M. Edkins, G. Clermont, A. N. Lazar, M. Haehnel, K. Edkins, A. Eichhorn, M. Blanchard-Desce, L. Meinel, T. B. Marder, *Chem. Eur. J.* **2016**, *22*, 14701-14706.

[3] S.-F. Liu, Q. Wu, H. L. Schmider, H. Aziz, N.-X. Hu, Z. Popović, S. Wang, *J. Am. Chem. Soc.* **2000**, *122*, 3671-3678.

[4] a) N. S. Makarov, M. Drobizhev, A. Rebane, *Opt. Express* **2008**, *16*, 4029-4047; b) S. de Reguardati, J. Pahapill, A. Mikhailov, Y. Stepanenko, A. Rebane, *Opt. Express* **2016**, *24*, 9053-9066.

[5] Gaussian 09, Revision D.01, M. J. Frisch, G. W. Trucks, H. B. Schlegel, G. E. Scuseria, M. A. Robb, J. R. Cheeseman, G. Scalmani, V. Barone, B. Mennucci, G. A. Petersson, H. Nakatsuji, M. Caricato, X. Li, H. P. Hratchian, A. F. Izmaylov, J. Bloino, G. Zheng, J. L. Sonnenberg, M. Hada, M. Ehara, K. Toyota, R. Fukuda, J. Hasegawa, M. Ishida, T. Nakajima, Y. Honda, O. Kitao, H. Nakai, T. Vreven, J. A. Montgomery, J. E. P. Jr., F. Ogliaro, M. Bearpark, J. J. Heyd, E. Brothers, K. N. Kudin, V. N. Staroverov, T. Keith, R. Kobayashi, J. Normand, K. Raghavachari, A. Rendell, J. C. Burant, S. S. Iyengar, J. Tomasi, M. Cossi, N. Rega, J. M. Millam, M. Klene, J. E. Knox, J. B. Cross, V. Bakken, C. Adamo, J. Jaramillo, R. Gomperts, R. E. Stratmann, O. Yazyev, A. J. Austin, R. Cammi, C. Pomelli, J. W. Ochterski, R. L. Martin, K. Morokuma, V. G. Zakrzewski, G. A. Voth, P. Salvador, J. J. Dannenberg, S. Dapprich, A. D. Daniels, O. Farkas, J. B. Foresman, J. V. Ortiz, J. Cioslowski, D. J. Fox, Gaussian, Inc., Wallingford CT, Gaussian, Inc., Wallingford CT, **2009**.

[6] a) A. D. Becke, *J. Chem. Phys.* **1993**, *98*, 5648-5652; b) C. Lee, W. Yang, R. G. Parr, *Phys. Rev. B* **1988**, *37*, 785-789; c) P. J. Stephens, F. J. Devlin, C. F. Chabalowski, M. J. Frisch, *J. Phys. Chem.* **1994**, *98*, 11623-11627.

[7] a) G. A. Petersson, M. A. Al-Laham, *J. Chem. Phys.* **1991**, *94*, 6081-6090; b) G. A. Petersson, A. Bennett, T. G. Tensfeldt, M. A. Al-Laham, W. A. Shirley, J. Mantzaris, *J. Chem. Phys.* **1988**, *89*, 2193-2218.

[8] T. Yanai, D. P. Tew, N. C. Handy, *Chem. Phys. Lett.* **2004**, *393*, 51-57.

[9] a) S. Jungsuttiwong, R. Tarsang, T. Sudyoadsuk, V. Promarak, P. Khongpracha, S. Namuangruk, *Organic Electronics* **2013**, *14*, 711-722; b) J. Preat, *J. Phys. Chem. C* **2010**, *114*, 16716-16725; c) P. Wiggins, J. A. G. Williams, D. J. Tozer, *J. Chem. Phys.* **2009**, *131*, 091101; d) M. J. G. Peach, P. Benfield, T. Helgaker, D. J. Tozer, *J. Chem. Phys.* **2008**, *128*, 044118; e) M. J. G. Peach, A. J. Cohen, D. J. Tozer, *Phys. Chem. Chem. Phys.* **2006**, *8*, 4543-4549.

[10] J. Tomasi, B. Mennucci, R. Cammi, *Chem. Rev.* **2005**, *105*, 2999-3094.

[11] a) T. Lu, F. Chen, *J. Comput. Chem.* **2012**, *33*, 580-592; b) R. L. Martin, *J. Chem. Phys.* **2003**, *118*, 4775-4777.
